# Triplet-Encoded Prebiotic RNA Aminoacylation

**DOI:** 10.1021/jacs.3c03931

**Published:** 2023-07-12

**Authors:** Meng Su, Christian Schmitt, Ziwei Liu, Samuel J. Roberts, Kim C. Liu, Konstantin Röder, Andres Jäschke, David J. Wales, John D. Sutherland

**Affiliations:** † MRC Laboratory of Molecular Biology, Cambridge CB2 0QH, UK; ‡ Institut für Pharmazie und Molekulare Biotechnologie, Universität Heidelberg, Heidelberg 69120, Germany; § Yusuf Hamied Department of Chemistry, University of Cambridge, Cambridge CB2 1EW, UK

## Abstract

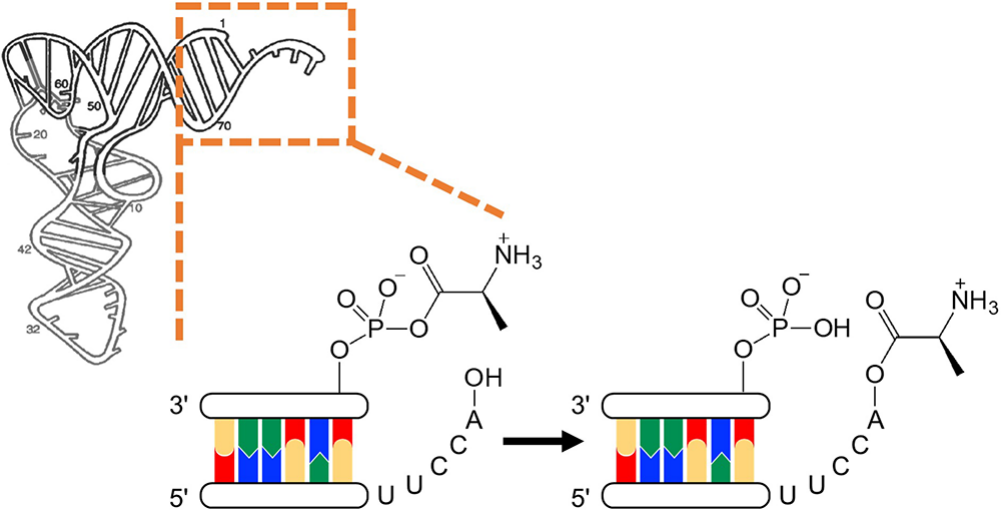

The encoding step of translation involves attachment of amino acids to cognate tRNAs by aminoacyl-tRNA synthetases, themselves the product of coded peptide synthesis. So, the question arises—before these enzymes evolved, how were primordial tRNAs selectively aminoacylated? Here, we demonstrate enzyme-free, sequence-dependent, chemoselective aminoacylation of RNA. We investigated two potentially prebiotic routes to aminoacyl-tRNA acceptor stem-overhang mimics and analyzed those oligonucleotides undergoing the most efficient aminoacylation. Overhang sequences do not significantly influence the chemoselectivity of aminoacylation by either route. For aminoacyl-transfer from a mixed anhydride donor strand, the chemoselectivity and stereoselectivity of aminoacylation depend on the terminal three base pairs of the stem. The results support early suggestions of a second genetic code in the acceptor stem.

## Introduction

The translation of genetic information contained in mRNA into specific protein sequences according to the genetic code depends on two molecular recognition events: first, attachment of specific amino acids to cognate tRNAs, and second, binding of charged tRNAs to mRNA. The latter depends primarily on anticodon:codon binding mediated by the nucleic acids themselves through Watson-Crick base pairing, with help from the decoding center of the ribosome. The attachment of amino acids to cognate tRNAs, on the other hand, does not depend on amino acid:RNA interactions, but has an obligate requirement for enzyme control. Specific aminoacyl-tRNA synthetases (aa-tRNA synthetases) recognize both amino acids and cognate tRNAs and catalyze their joining together in an ATP-consuming reaction.^1^ Two questions relating to the origin of translation thus arise: “how could specific aminoacylation of cognate tRNAs have been achieved without enzymes?” and “on what basis were amino acid:codon assignments initially made?”.

The limited experimental work bearing on these questions has left answers mainly in the realm of conjecture.^2^ The role of the stereochemical interaction between amino acids and tRNA in setting the genetic code has been considered by several authors with opinions varying as to where the tRNA residues involved in the recognition reside.^3−6^ In principle, such stereochemical interactions could answer both questions at once, if amino acid:anticodon interactions simultaneously controlled both specificity of aminoacylation and amino acid:codon assignment. However, there has been no clear experimental support for this assumption for many decades, so the possibility of stereochemical interactions with RNA residues other than the anticodon remains open, but it is difficult to see how this could have affected codon assignment.^7^ The fact that RNA enzymes (ribozymes) can catalyze uncoded aminoacylations, and also, peptide couplings in the absence of proteins has been amply demonstrated.^8−12^ However, these systems do not allow conclusions to be drawn about codon-amino acid assignments. The “frozen accident theory” has it that assignments were made at random and then became fixed to maintain genotype:phenotype integrity.^13^ Alternatively, it has been argued that assignments were made to minimize the phenotypic effect of coding errors or were made sequentially as new amino acids became available through biosynthesis.^14^

The realization that tRNA identity determinants—used by aminoacyl-tRNA synthetases to determine whether a tRNA is cognate—do not always include the anticodon resulted in a major conceptual advance.^15,16^ It was found that many identity determinants are clustered in the tRNA acceptor stem,^17^ indeed in one case, identity is determined by a single base pair in the acceptor stem.^18−20^ This led to the suggestion that a “second genetic code” is written in the acceptor stem of tRNA and read by aa-tRNA synthetases.^21^ It was speculated that this “still largely undeciphered” second code might be older and more deterministic than the classical genetic code, possibly even depending on stereochemical interactions between a particular sequence in the acceptor stem and the cognate amino acid or aminoacyl-intermediate^22−25^ although other views exist.^26,27^ Short RNA molecules (aptamers) selected from random sequence mixtures by amino acid binding have been reported to be enriched with cognate triplets for the respective amino acids,^28^ but the studies have not been extended to chemical reactions of the bound amino acids. In the absence of enzymes, production of selectively aminoacylated tRNAs would require aminoacylation to somehow be coded by RNA sequence.

**Figure 1:**
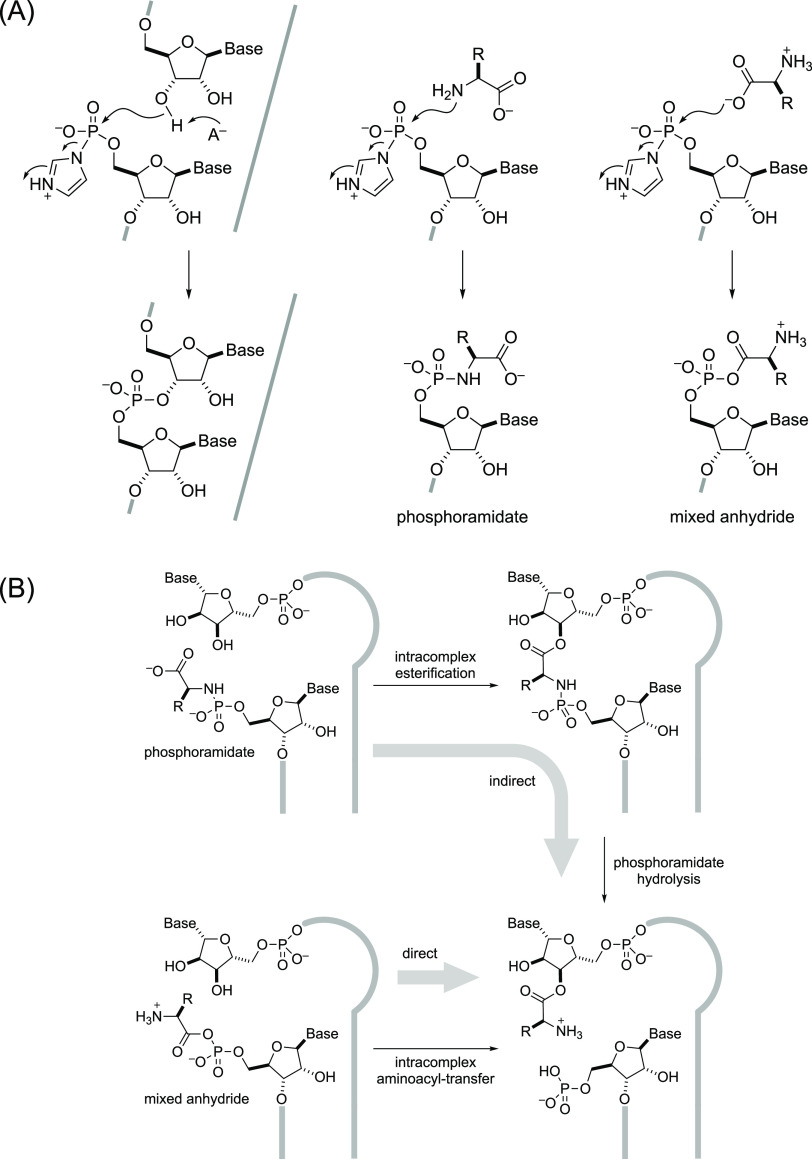
RNA aminoacylation via activated phosphates. (A) Activated phosphates required for RNA ligation chemistry also react with amino acids to give phosphoramidates and/or mixed anhydrides depending on pH; (B) production of 3′-aminoacyl-RNA by indirect and direct transfer of aminoacyl groups from phosphoramidates and mixed anhydrides, respectively.

Several years ago, we uncovered a “protometabolic” network of reactions based on the reductive homologation of hydrogen cyanide and its derivatives by hydrogen sulfide under photochemical conditions.^29^ This “cyanosulfidic” chemistry led to precursors of nucleotides as well as amino acids suggesting that the assembly into higher-order structures occurred in mixtures of these building blocks. The activated nucleotides and oligonucleotides required to assemble RNA by sequential monomer addition and ligation, respectively, are known to undergo competing reactions with amino acids that depend on pH.^30^ In a mildly alkaline solution, reaction of the free amino groups with activated nucleotides results in phosphoramidates, whereas under slightly acidic conditions, where amino groups are substantially protonated, the carboxylate groups attack instead, affording mixed carboxylic-phosphoric anhydrides^31−33^ (Figure 1A). We recently discovered efficient chemistries, whereby an amino acid is transferred from either sort of RNA:amino acid conjugate at the 5′-phosphate of a tRNA acceptor stem mimic to the 2′,3′-diol terminus of a short 3′-overhang^34,35^ (Figure 1B). In light of the aforementioned suggestions of a second genetic code, we wondered if there might be a relationship between the sequence of the acceptor stem-overhang and which amino acid is most efficiently transferred by one or other chemistry.

## Results and Discussion

Based on our earlier work on mixed anhydride chemistry, we varied the nature of the 3′-overhang attached to an acceptor stem duplex mimic and used HPLC to monitor the efficiency of direct interstrand aminoacyl-transfer. Through a very sparse sampling of aminoacyl residues and overhang length and sequence (Tables S1 and S2), we found that the five base overhang 5′-UUCCA-3′ (overhang sequences underlined) allowed the most efficient and stereoselective (l- preferred over d-)alanyl-transfer. We then used this same overhang sequence to investigate stereoselectivity and the effect of changing amino acids on indirect aminoacyl-transfer starting from phosphoramidates and proceeding via phosphoramidate-ester intermediates. Stereoselectivity (l- preferred overd-) was maintained for this indirect aminoacyl-transfer across a range of amino acids, but yields were amino acid-dependent (Table S3). To extend our earlier investigations, we fully randomized a five-base overhang (Table S4) and selected those sequences best able to undergo direct or indirect aminoacyl-transfer using either chemistry at the 5′-terminus of a tRNA acceptor stem mimic. Within the subset of amino acids whose aminonitrile precursors can be made by cyanosulfidic chemistry,^29^ for ease of synthesis, we further restricted ourselves to making glycyl-, l-/d-alanyl, l-prolyl-, l-leucyl-, and l-valyl-mixed anhydrides and phosphoramidates of 5′-pAGCGA-3′ and separately subjected them to aminoacyl-transfer to an annealed pool of oligonucleotides with the 3′-terminal decanucleotide sequence: 5′-UCGCUNNNNN-3′. In the case of indirect aminoacyl-transfer, we had previously shown that the second step (mild acid hydrolysis, Figure 1B) proceeds in a uniformly high yield with the amino acids we had chosen.^35^ For this reason, as well as for experimental practicality, we did not hydrolyze the phosphoramidate P–N bond to form an aminoacyl-ester in the current work.

To determine which sequences had undergone the most efficient direct aminoacyl-transfer, or phosphoramidate-ester formation with the two chemistries, we developed a high-throughput screen strategy (Figure S1). Acylation of an RNA 2′,3′-diol terminus protects it against oxidation by periodate, whereas free 2′,3′-diol termini are oxidized to nonligatable dialdehyde termini. Subsequent hydrolysis of aminoacyl-ester termini and bridged phosphoramidate-esters allowed ligation of the newly liberated diol termini to an adapter oligonucleotide complementary to a reverse transcription primer. Successful ligation enabled reverse transcription and PCR amplification using primers adapted to multiplex sequencing (Table S4). Next-generation sequencing of the amplified sequence mixture then allowed us to find those overhang sequence variants that gave the most efficient aminoacyl-transfer or phosphoramidate-ester formation (by analyzing the number of sequencing reads for all 1024 pentanucleotides) and to rank them for each amino acid. As a blank, we subjected the partially randomized oligonucleotide pool only to the ligation, reverse transcription, and PCR amplification and sequenced it to reveal those variants favored by this sample processing procedure. The tabulated data for the aminoacyl-transfer and phosphoramidate-ester formation experiments and the blank were visualized to display the sequence preferences in a graphical format and compared them in scatter plots to discern whether the preferred sequences for one aminoacyl residue were systematically related to those of any other (Figure S2). For direct aminoacyl-transfer from mixed anhydride RNA:amino acid conjugates, the results revealed that different aminoacyl residues are transferred to a similar subset of U, G-rich overhang sequences and that this subset of sequences is different from the U, A-rich subset that is favored by the blank sample processing procedure. This demonstrates that certain overhang sequences enable more efficient aminoacyl-transfer than others. However, crucially, there appears to be no idiosyncrasy with respect to the transfer of specific aminoacyl residues. We found that the 5′-UUCCA-3′ sequence (previously determined from sparse sampling to undergo efficient l-alanyl-transfer) was among the top 10% of sequences for all amino acids tested and, in each case, was the best among sequences ending in the 5′-CCA-3′ trinucleotide sequence (Tables S5 and S6). For phosphoramidate-ester formation, A-deficient overhang sequences were preferred across the range of amino acids, while the blank preference was U, A-rich. Thus, neither chemistry was associated with significant selective preference of different overhang sequences for different amino acids—no hints of coding were apparent. Our attention was thus switched to the stem region. We stuck with 5′-UUCCA-3′ because it was favored in the mixed anhydride chemistry, reasonably efficient in the phosphoramidate-ester chemistry, and close to the canonical tRNA overhang sequence.

**Figure 2:**
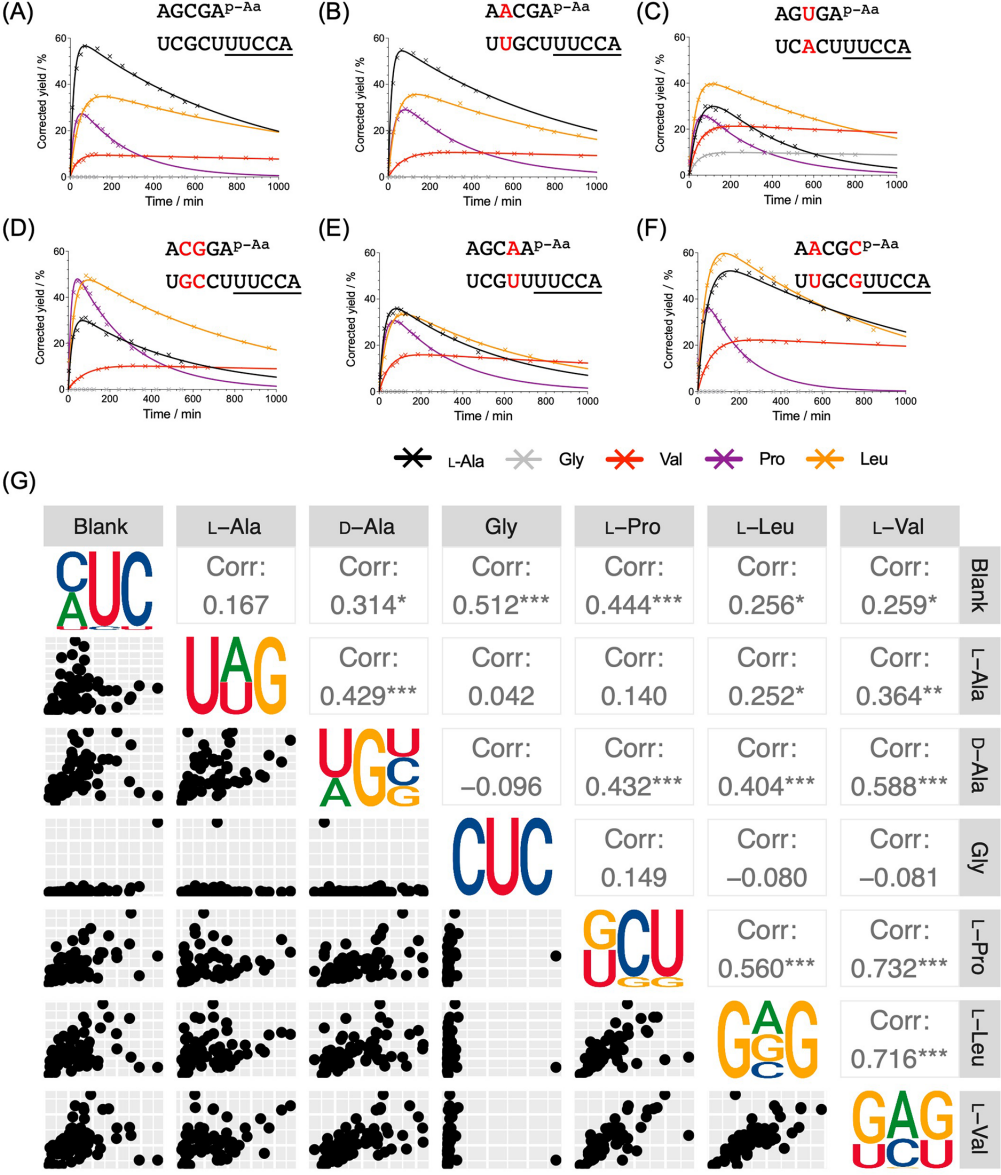
Divergent patterns in the first three nucleotides in the stem. (A–F) Time courses showing the aminoacyl-transfer kinetics with identical overhang “UUCCA”, but base pairs changed in the stem (indicated by the red font); solid lines represent the best fits from nonlinear regression analyses. (G) Plot of sequencing results after mixed anhydride aminoacylation selection using a partially randomized stem (5′-GAUUCNNNUUCCA) showing correlation between amino acids. Sequence logo represents the top 10% of trinucleotides from the raw reading numbers. Axes of the scatter plots are read numbers and are omitted for simplicity. Pearson correlation coefficients are shown in the upper right corner. Aminoacyl-transfer conditions: both oligos (100 μM), NaCl (100 mM), MgCl_2_ (5 mM), HEPES (50 mM, pH 6.8).

We focused on the effect of stem sequence on the mixed anhydride chemistry first. Unsure of how many stem residues to randomize, we synthesized a range of oligonucleotide donor-acceptor pairings with base pair changes at specific positions to delineate the region of the stem, if any, affecting the specificity of aminoacyl-transfer. These oligonucleotide combinations were then analyzed in an HPLC-based kinetic assay (Tables S1 and S7–S12, ref (34)). In this way, we found that the three base pairs of the stem proximal to the overhang influenced aminoacyl-transfer (Figure 2A,C–F), whereas changing the more distal fourth base pair had no effect (Figure 2B). We, therefore, randomized the three residues upstream of the 5′-UUCCA-3′ overhang of an acceptor oligonucleotide (3′-terminal tridecanucleotide sequence: 5′-GAUUCNNNUUCCA-3′) to be used with a randomized octanucleotide donor (5′-pNNNGAAUC-3′, Figure S3). After annealing of separate aminoacyl-phosphate mixed anhydrides of the donor oligonucleotide pool randomized in the three 5′-residues, we submitted the samples to aminoacyl-transfer conditions and sequenced those acceptor strands that were protected from periodate oxidation by aminoacyl-transfer. Again, we performed a blank in which solely the partly randomized acceptor oligonucleotide was subjected to sample processing. Sequencing revealed that, for some amino acids, those sequence variants that underwent the most efficient aminoacyl-transfer were very different, whereas for others, there were similarities (Figure 2G, Tables S13–S15). Thus, for example, the preferred sequences for l-alanyl-, d-alanyl-, and glycyl-transfer were very different from each other and from the sequences preferred for transfer of other aminoacyl residues (as apparent from the comparison of the graphic representation of the top 10% of reads and from scatter plots in which a systematic relationship would have resulted in points clustered on a diagonal). On the other hand, there was a clear similarity between those sequences preferred for l-valyl- and l-leucyl-transfer and a lesser similarity between those sequences and the sequences preferred for l-prolyl-transfer. Glycyl-transfer stood out in the sense that the results were dominated by a single sequence (5′-CUC-3′), which appeared in 44% of reads. Ignoring this highly represented sequence, the remaining preferences were still different from those for transfer of other aminoacyl residues (Figure S4). In no case did the trinucleotide preferences for transfer of any particular aminoacyl residue bear any obvious resemblance to the extant codon or anticodon sequences for the corresponding amino acid. So, it appears unlikely that this stereochemical coding is connected to codon assignment. However, it is consistent with a “second genetic code” in the acceptor stem.^21^ This second code relates to the specificity of tRNA aminoacylation, but not directly to codon assignment.

We then investigated the effect of the stem sequence on the phosphoramidate-ester chemistry. To allow comparison to the results for the mixed anhydride chemistry, we randomized the same trinucleotide region of the stem and separately annealed portions of this library to specific amino acid phosphoramidates of the complementary randomized donor strand (Figure S5). After subjecting the various acceptor stem-overhang combinations to esterification conditions, we then used the same procedure that we had used before. In contrast to what we had seen with the mixed anhydride chemistry, sequencing revealed that the nature of the amino acid had almost no effect on the preferred sequences for phosphoramidate-ester formation, although the consensus sequence preference was distinctly different from the preferences seen for the blank sample (Figure S6, Tables S16 and S17). We felt that the complete lack of amino acid side chain influence on the preferred stem sequences for phosphoramidate-ester formation made the idiosyncrasies we had seen with the mixed anhydride chemistry even more remarkable. Accordingly, we now abandoned the indirect aminoacyl-transfer chemistry and focused exclusively on the direct transfer chemistry.

**Figure 3:**
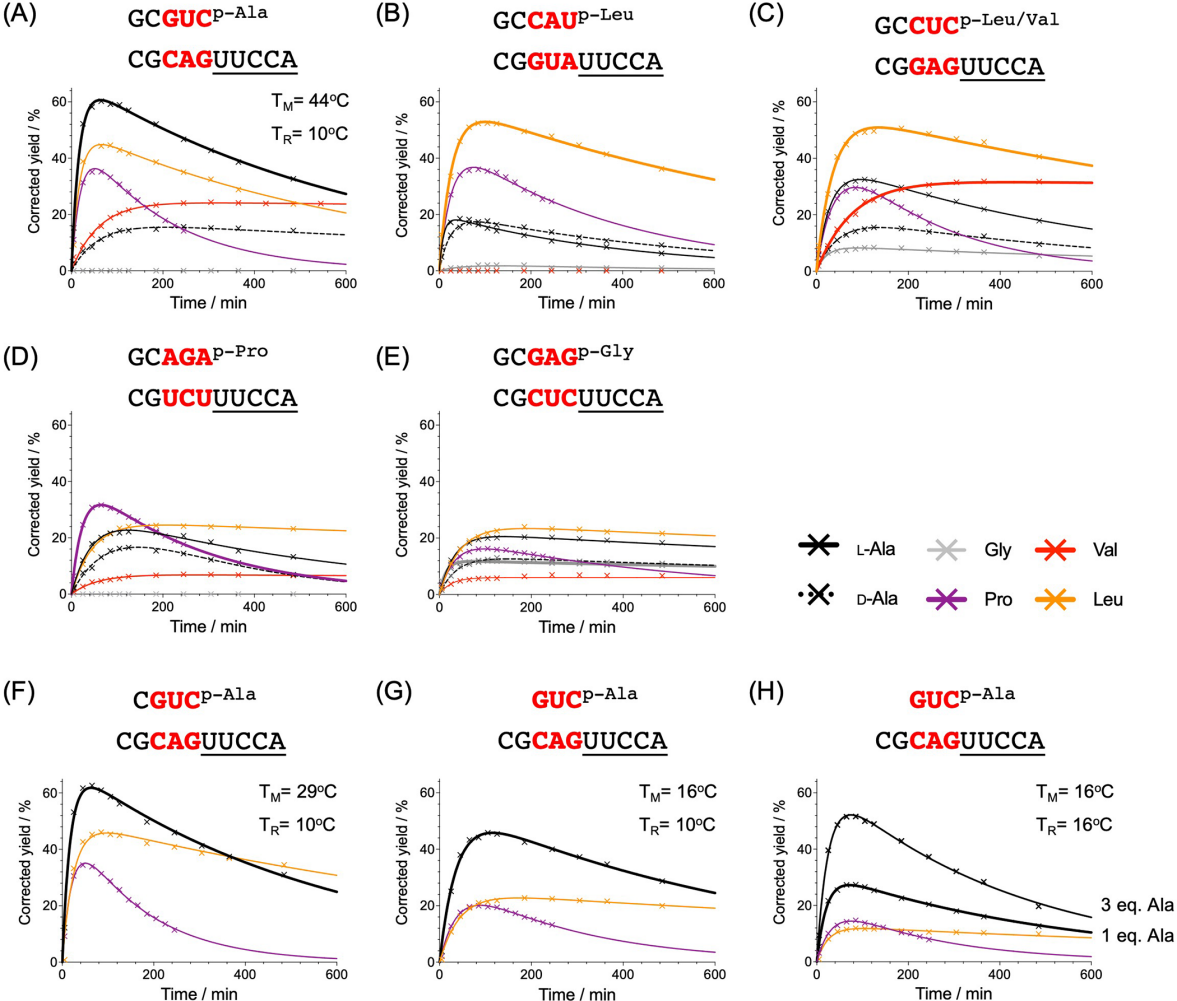
Stereochemical coding dictated by a stem-terminal sequence. Time courses showing the mixed anhydride aminoacyl-transfer kinetics with varied trinucleotide stem sequences according to the top selectivity score (A) l-Ala; (B) l-Leu; (C) l-Leu and l-Val; (D) l-Pro; (E) Gly, (A–E) all with 1 equiv of donor transferred at 10 °C; (F) 1 equiv of tetramer donor for l-Ala at 10 °C; (G) 1 equiv of trimer donor for l-Ala at 10 °C; (H) 1 or 3 equiv of trimer donor for l-Ala at 16 °C. *T*_M_, melting temperature; *T*_R_, temperature at which the aminoacyl-transfer reaction was conducted. Solid lines represent the best fits from nonlinear regression analyses. Conditions: both oligos (100 μM), NaCl (100 mM), MgCl_2_ (5 mM), HEPES (50 mM, pH 6.8).

The selection and sequencing protocol cannot be expected to give quantitative data about the kinetics of aminoacyl-transfer. Accordingly, we used the results to guide our choice of sequences for the determination of the kinetics of individual aminoacyl-transfer reactions by HPLC. As sequencing only determined the acceptor strand, for the kinetic analysis, we paired it with its Watson-Crick complement in most cases. The first such pairing was based on a trinucleotide combination that the sequencing data suggested should give efficient and selective aminoacylation with l-Ala (acceptor 5′-CAG-3′ and its complement 5′-CUG-3′). Indeed, l-Ala was more rapidly and completely converted in the HPLC assay than any of the other amino acids tested (Figure 3A, Table S18).

Remarkably, glycyl-transfer between the same oligonucleotides was undetectable. The stereoselectivity of alanyl-transfer was high (l- preferred over d- by a factor of 3.5, Table S18), and although l-prolyl, l-valyl, and l-leucyl residues were also transferred fairly well, product yields were not as high as they were with l-alanine. The principal alanyl-tRNA identity determinant in extant biology is the G3:U70 wobble base pair (although a C3:G70 base pair is also functional).^18,19,36^ We investigated the effect of a similar wobble base pair on l-alanyl-transfer in our system by keeping the same donor strand and changing the acceptor to one terminating in the sequence 5′-UAG-3′ (Table S18). The change to a wobble base pair slowed down l-alanyl-transfer and almost halved the maximum yield of the transfer product.

According to the sequencing data, the general trinucleotide preferences for l-leucyl and l-valyl transfer are similar. However, the kinetic analysis of the transfer of these more hydrophobic amino acids revealed that the 5′-GUA-3′ trinucleotide is efficient in the transfer of leucine but inefficient in the transfer of valine (Figure 3B, Table S19). In contrast, the trinucleotide 5′-GAG-3′, which sequencing had suggested as the consensus sequence for both l-leucyl- and l-valyl-transfer, transfers both amino acids efficiently. In this system, glycyl transfer is now observed, albeit in a lower yield (Figure 3C, Table S20). The top-ranked sequence for proline transfer, 5′-UCU-3′, was found to transfer l-proline more efficiently than all other amino acids in the HPLC assay (Figure 3D, Table S21). The sequencing data further suggest that l-valyl-transfer should be more efficient than l-leucyl-transfer with the sequence 5′-GCU-3′ (Table S14). However, we had already investigated that sequence when we were delineating which part of the stem affects aminoacyl-transfer—albeit with a stem differing at the fourth and fifth positions—and found the opposite to be the case (Figure 2A,B). Thus, as is generally held in the field although the sequencing data can serve as a guide to discern selectivity, it cannot be relied upon to give quantitative data.

For many stem-terminal trinucleotide sequences, glycyl-transfer was barely detectable. However, as mentioned above, low yield transfer was apparent for the trinucleotide 5′-GAG-3′ (Figure 3C). Using the top-ranked trinucleotide 5′-CUC-3′, glycyl-transfer was somewhat more efficient, but was still less efficient than l-leucyl- and l-prolyl- and alanyl-transfer (Figure 3E, Table S22). If the abundances of prebiotic amino acids on early Earth were largely equal, then the implication is that selectively glycylated tRNAs could not likely have been produced by stereochemical coding. However, given its simplicity, glycine was likely the most abundant prebiotic amino acid.^29,37,38^ If its concentration was significantly higher than any of the other amino acids, then stem-overhang pairings based on the trinucleotide 5′-CUC-3′ could have been selectively glycylated while glycylation of other stem-overhangs might have been less efficient than their aminoacylation with other amino acids. Thus, with a restricted subset of amino acids, stereochemical coding dictated by the stem-terminal trinucleotide sequence is possible. Furthermore, high stereoselectivity (l- preferred over d-) was also imposed on alanyl-transfer using the stem-terminal trinucleotide 5′-CAG-3′ and its complement 5′-CUG-3′ (Figure 3A). Several other oligonucleotide donor-acceptor pairings were associated with similarly stereoselective alanyl-transfer (e.g., Figure 3C), but the donor-acceptor pair that underwent efficient l-leucyl-transfer (stem-terminal trinucleotide 5′-GUA-3′ and its complement 5′-UAC-3′, Figure 3B) displayed almost no stereoselectivity for alanyl-transfer. Stereoselectivity, like amino acid chemoselectivity, is thus controlled by the stem-terminal trinucleotide sequence.

In extant biology, tRNAs are aminoacylated using aminoacyl-adenylates, which are much smaller than the donor oligonucleotides used here. Therefore, we next investigated if stereochemical coding is maintained in our system when the length of the oligonucleotide donor is progressively reduced. For the duplex of a nonaminoacylated pentanucleotide donor and decanucleotide acceptor (5′-CGCAGUUCCA-3′), we determined a melting temperature *T*_M_ of 44 °C. This value is well above the 10 °C temperature at which we carried out the aminoacyl-transfer reactions, resulting in the formation of a stable duplex under the assay conditions (Figure 3A and Figure S7). Truncating the donor to a tetranucleotide while keeping the acceptor constant lowered the *T*_M_ to 29 °C, and the aminoacyl-transfer efficiency remained almost unchanged for the three amino acids studied (compare Figure 3A,F). Using a trinucleotide donor with the *T*_M_ of 16 °C reduced the efficiency of transfer of all three amino acids, but increased in the selectivity of coding for l-alanyl-transfer (Figure 3G and Table S23). At a *T*_M_ so close to the reaction temperature, a significant portion of the strands is present dissociated, and we found that the yield of the aminoacylated acceptor strand was markedly increased when we used three equivalents of the l-alanyl donor strand (Figure 3H). Ribozyme auxiliaries could then improve the aminoacylation selectivity resulting in better coding and progressively allow the use of aminoacylated di- and mono-nucleotides. Thus, a series of small steps can be envisaged to take the chemistry that we have uncovered here to the point where coded peptides could start to contribute to the efficiency of their own generation through becoming aminoacyl-tRNA synthetases.

While there is clear evidence for sequence and aminoacyl side chain dependence in the transfer selectivity, it is not clear how this selectivity arises. One possibility is that differences in the structures adopted by the different sequences and aminoacyl groups may play a key role in determining the observed trends. To investigate this possibility further, we conducted simulations exploring the underlying energy landscapes to identify whether such structural changes exist. The first set of calculations focused on two distinct sequences each for l-alanyl and l-leucyl transfer, one with a high, and one with a lower, yield and *k*_transfer_ (Figure S8 and Tables S24 and S25). We found that the structural ensembles indeed exhibit distinct characteristics. In both cases, the lower-yielding sequence (Figure S8B,D) makes many close contacts between the amino acid and atoms of the oligonucleotide duplex (red spikes in the energy landscapes) that the higher-yielding counterpart (Figure S8A,C) does not make. These interactions are either between the Hoogsteen edges of the second and third base pair with either the phosphate group linking the aminoacyl group to the nucleic acid (Figure S8 snapshots B1and B2) or the amino group of the amino acid (Figure S8 snapshots D1–D3). The selectivity in these scenarios arises for the first set (Figure S8A,B) of interactions from the nature of the Hoogsteen edge (an available electron-rich O or N for the amide or a OH or NH for the phosphate), and in the second case from the ability to rearrange the backbone when there is weaker base pairing at the top of the stem (A:U vs C:G, Figure S8C,D). These contacts make a close approach of the overhanging 3′-end of the acceptor strand to the aminoacyl group geometrically challenging, suggesting an energy barrier for the transfer, as these contacts need to be lost first. This effect likely lowers the yield, but the contacts are not strong enough to completely suppress the reactions.

**Figure 4:**
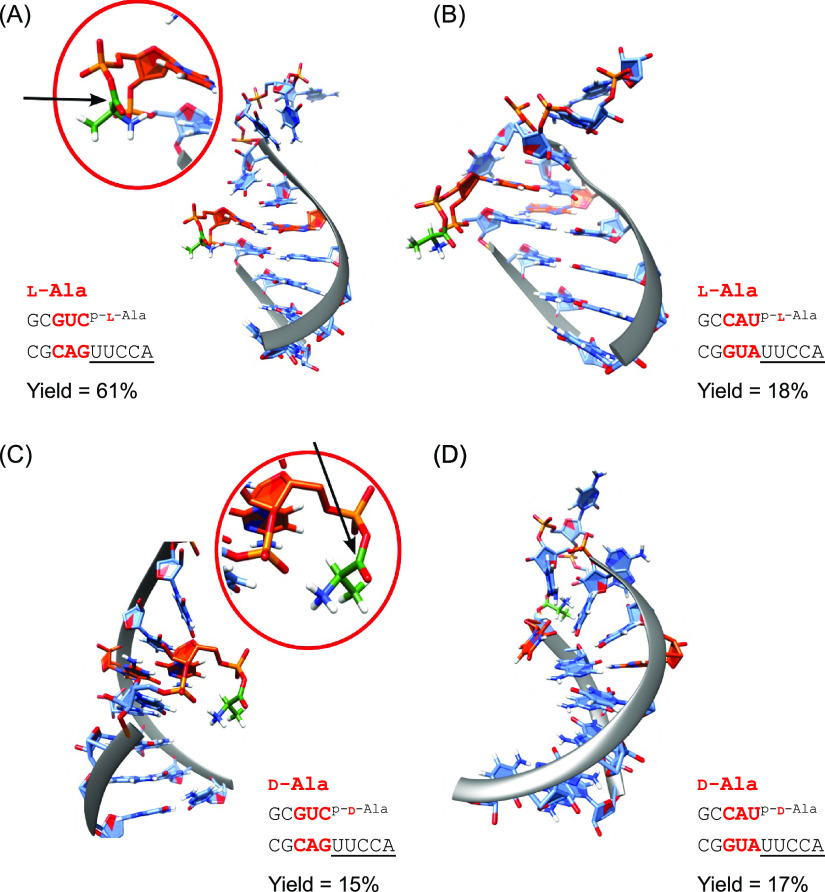
Representative structures for l-Ala (A, B) and d-Ala systems (C, D), selected from the energy landscape databases. Comparing panels A and C suggests an explanation for the observed stereoselectivity. For l-Ala (A), the Burgi-Dunitz trajectory is freely accessible (black arrow). In contrast, the trajectory is blocked in d-Ala (C). For panels B and D, the top base pair in the stem is frayed in both cases, giving more flexible structures. The aminoacyl moiety is highlighted in green, and the top pair of bases in the stem is highlighted in orange.

Once we had diagnosed a structural basis for the observed difference in yields, a second set of simulations focused on the systems in Figure 3A,B to determine whether the stereoselectivity for alanyl-transfer, the difference in yields for l-valyl- and l-leucyl-transfer, and the low yield for glycyl-transfer can also be linked to structural features (Figure 4 and Figure S9). For the sequence from Figure 3A, the top base pair is G:C and always intact, while for the systems from Figure 3B, the A:U base pair is absent in the case of valine, alanine, and glycine.

For the l-valyl-mixed anhydride, the loss of base pairing leads to strong interactions of aminoacyl group and the distorted stack, which forms a triplet. As a result, the overhang is not able to achieve the proximity required for the transfer (Figure S9C,E,F). In contrast, the l-leucyl-stem-overhang exhibits no such distortions and is accessible for the transfer reaction (Figure S9A,B,E). We speculate that the difference in behavior arises from the side chain length, and the increased hydrophobicities, as the glycyl-, alanyl-, and valyl-stem-overhangs, all exhibit the change in the stem, but the leucyl-stem-overhang does not.

The stereoselectivity between l- and d-alanyl-transfer is also linked to the stability of the top base pair in the stem. A stable base pair allows interactions of the alanyl residue with the stack, but in such a fashion that the 3′-overhang is able to get in close proximity. However, while the l-stereoisomer enables access to the carbonyl carbon along the Burgi-Dunitz trajectory,^39,40^ the d-stereoisomer, as the interactions with the nucleobases are the same, is flipped and the approach to the carbonyl is blocked by the stem (Figure 4A,C). When the top base pair is lost, these configurations are no longer observed, and the selection bias disappears (Figure 4B,D).

Finally, the glycyl residue in all cases exhibits strong interactions, likely stabilized by the absence of a hydrophobic side chain.

## Conclusions

The attractiveness of tRNA self-aminoacylation as a prelude to coded translation was alluded to over 50 years ago.^13^ Our finding that triplet-encoded chemo- and stereoselective tRNA acceptor stem-overhang mimic aminoacylation is possible and thus provides the first experimental support for these earlier suggestions. The function of the first coded peptides is not known, nor is the precision of coding required to enable this function. The degree of coding chemo- and stereoselectivity that we have discovered is not particularly high, but it might have resulted in the loosely coded synthesis of short peptides composed predominantly of l-amino acids. It would also have been something that nascent biology could have built on. Thus, ribozymes could have evolved to enhance this intrinsic chemical coding. These ribozymes could then have assisted the aminoacyl-transfer from progressively shorter oligonucleotide donors, ultimately resulting in the transfer from aminoacyl-adenylates. The chemistry, involving direct transfer to the 2′,3′-diol from a mixed anhydride of the amino acid and the 5′-phosphate, resembles the second step of the chemistry catalyzed by aminoacyl-tRNA synthetases in extant biology. Concomitant improvements in chemo- and stereoselectivity along with assignment to codons and development of translation could then lead to the synthesis of peptides sufficiently well encoded that they could augment the function of the ribozymes and, ultimately replace them.

Our original choice of stem-overhang was based on a model for the origin of tRNA by direct gene duplication.^34,41^ The ligation junction required by this model is situated in the anticodon loop, and we postulated that an overhang sequence adopting a folded-back conformation would be most prone to such a ligation. Symmetry then dictated that what would have been destined to become the acceptor overhang would also have adopted a folded-back conformation.^42^ Based on this model, we then used both canonical extant acceptor stem-overhang sequence preferences and the corresponding anticodon loop sequence preferences in the design of the putative ancestral overhang. This resulted in a five-base overhang, which is shorter than the extant anticodon loop,^43,44^ but longer than the extant acceptor overhang. We then showed experimentally that the folded-back overhang indeed allows both aminoacyl-transfer from the 5′-phosphate to the 2′,3′-diol and loop-closing ligation.^45^ The pentanucleotide overhang and often frayed acceptor stem terminus that allow coded aminoacylation by purely chemical means would not be expected to remain unchanged as ribozyme- and then enzyme-catalyzed aminoacylation emerged. Indeed, predominant adoption of a G1:C72 base pair, replacement of the U responsible for the U-turn motif, and truncation of the overhang would enable a more rigid acceptor stem-overhang, which could project into a catalyst active site. So, extant tRNA acceptor stem overhang structures might only bear passing resemblance to ancestral structures. However, the coding by the terminal trinucleotide of the tRNA mimic provides a plausible explanation as to why modern tRNA identity determinants now cluster at the acceptor stem as well as at the anticodon.

The earliest aminoacyl-tRNA synthetase enzymes likely could not span the long distance (∼75 Å in extant tRNAs) from the site of acceptor stem aminoacylation to the anticodon, but could easily recognize RNA in the vicinity of the aminoacylation site. If the enzymes originally evolved to build on the intrinsic chemical coding by the acceptor stem,^46^ they would be expected to use the trinucleotide or elements thereof in the process of cognate tRNA recognition. The position of these identity determinants within the tRNA molecule would be expected to be retained even if their sequence identity was changed over time.

## Data Availability

All raw sequencing data and code for data cleaning and analysis associated with the current submission is available in a zenodo repository at 10.5281/zenodo.7515305. All simulation data is available at 10.5281/zenodo.7371596.

## Supporting Information Available

Chemical synthesis of RNA oligomers, chemical synthesis and characterization of protected amino acids, chemical synthesis of oligoribonucleotide:amino acid mixed anhydrides, aminoacyl-transfer from a mixed anhydride donor to the 3′-terminus of an acceptor overhang, calculations of the corrected yields, HPLC quantification and kinetic regression analysis, preparation of RNA aminoacyl-transfer library, free diol oxidation, ligation, reverse transcription, phosphoramidate chemistry, melting temperature measurement using MicroCal VP-Capillary DSC, and structure simulation (PDF)
