# Supplementary material for: Triplet-Encoded Prebiotic RNA Aminoacylation
Source: J Am Chem Soc. 2023 Jul 12;145(29):15971–80. doi: 10.1021/jacs.3c03931 (PMC10375532; doi:10.1021/jacs.3c03931)
Supplement: Supplementary file 1 — ja3c03931_si_001.pdf [file ja3c03931_si_001.pdf]

Supporting Information for

Triplet-encoded prebiotic RNA aminoacylation

Meng Su[1]†, Christian Schmitt[2]†, Ziwei Liu[1], Samuel J. Roberts[1], Kim C. Liu[1], Konstantin Röder[3**], Andres Jäschke[2*], David J. Wales[3*], John D. Sutherland[1*]

Corresponding author: John D. Sutherland, Andres Jäschke, David J. Wales

Email: johns@mrc-lmb.cam.ac.uk, jaeschke@uni-hd.de, dw34@cam.ac.uk

Materials and Methods

Reagents and solvents were obtained from *Acros Organics*, *Santa Cruz Biotechnology*, *Sigma-Aldrich*, *Synthon Chemicals GmbH & Co. KG* and *VWR International*, and were used without further purification. For solid-phase RNA synthesis, primer Support 5G with a loading of 300 µmol/g was purchased from *GE Healthcare*. Phosphoramidites for RNA synthesis were purchased from *Sigma-Aldrich* or *Link Technologies*. RNA oligomers in Table S1 and **Lib-S3-D** were synthesized using an ÄKTA Oligopilot plus 10 (*GE Healthcare*) on a 20 to 50 µmol scale. All other RNA oligomers in Table S3 were purchased from *Sigma-Aldrich* with HPLC purification. The purity of purchased oligomers were not checked before used.

A *Mettler Toledo* SevenEasy pH Meter S20 combined with a *ThermoFisher Scientific* Orion 8103BN Ross semi-micro pH electrode was used to measure pH during adjustment to the desired value. ^{1}H-, ^{13}C-, and ^{31}P-nuclear magnetic resonance (NMR) spectra were acquired using a *Bruker* Ultrashield 400 Plus or *Bruker* Ascend 400 operating at 400.13, 100.62, and 161.97 MHz, respectively. The notations s, t, and br represent the multiplicities of singlet, triplet, and broad signal, respectively. Chemical shifts (δ) are shown in ppm. Chemical shifts for P-NMR are referenced to 85% phosphoric acid, which is assigned the chemical shift of 0. Mass spectra were acquired on an *Agilent* 1200 LC-MS system equipped with an electrospray ionization (ESI) source and a 6130 quadrupole spectrometer. LC solvents: A, 0.2 % formic acid in H_2O – B, and 0.2 % formic acid in acetonitrile. Analytical High-Pressure Liquid Chromatography (HPLC) was run on Dionex Ultimate 3000 (*Thermo Scientific*) using a *Waters* A*tlantis* T3, 3 µm, 4.6 × 150 mm column. LC solvents: A, 20 mM triethylammonium acetate aq., pH = 7.0, and B, acetonitrile. Preparative HPLC were run on *Varian* PrepStar using a *Waters* Altantis T3 Prep OBD, 5 µm, 15 × 250 mm column. LC solvents: A, 100 mM triethylammonium acetate aq., pH = 7.0, and B, acetonitrile. Oligonucleotide concentrations were determined by UV absorbance at 260 nm using a *NanoDrop* ND-1000 spectrophotometer.

Chemical synthesis of RNA oligomers

After automated synthesis, RNAs were first cleaved from the solid support by treating with 3 mL of a 1:1 mixture of NH_3 aqueous solution (28% wt) and CH_3NH_2 ethanol solution (33% wt) at 55°C for 80 minutes in a tube with a sealed cap. The solid was removed by filtration and washed with 50 % EtOH/H_2O. The solutions were combined and evaporated to dryness under reduced pressure. Silyl protecting groups were removed by treating the residues with 2 mL of a 1:1 mixture of triethylamine trihydrofluoride and DMSO at 65°C for 150 minutes in a tube with a sealed cap. After brief cooling at -32°C, 40 mL of cold 50 mM $NaClO_4$ in acetone was added to the solution to precipitate the oligoribonucleotides. The resulting mixture was centrifuged and the recovered oligoribonucleotides purified by preparative HPLC followed by lyophilization. The purified RNA was redissolved in 2 mL of water and passed through a *Waters* Sep-Pak C18 Cartridge with 10 g sorbent.The cartridge was pre-washed with 50 mL of acetonitrile then 50 mL of water before sample loading, washed with 100 mL of H_2O and 50 mL of 20 % aqueous acetonitrile. Eluates were checked for RNA content using a NanoDrop spectrophotometer, checked for purity using analytical HPLC, combined and lyophilized. The resulting white powder was stored at -32°C for future use.

Mixed anhydride chemistry

Chemical synthesis of protected amino acids

Synthesis of *N*-4-monomethoxytritylvaline (MMTr-Val-OH), *N*-tritylleucine (Trt-Leu-OH), and *N*-tritylproline (Trt-Pro-OH)

Trimethylsilyl chloride (1.27 mL, 10 mmol, 1 eq.) was added to a magnetically stirred suspension of the amino acid (Val, Leu, or Pro, 10 mmol, 1 eq.) in chloroform/acetonitrile (18 mL, v/v 5/1) at room temperature. The reaction mixture was heated under reflux for 2 h and then allowed to cool to room temperature. A chloroform solution (10 mL) of triethylamine (2.79 mL, 20 mmol, 2 eq.) and 4-monomethoxytrityl chloride or trityl chloride (10 mmol, 1 eq.) was added to the mixture. The reaction was further stirred at room temperature overnight before the addition of methanol (2 mL, 50 mmol). The resulting mixture was evaporated to dryness and the residue was treated with a mixture of diethyl ether (50 mL) and a pre-cooled aqueous solution of citric acid (5%, 50 mL). The organic phase was separated and washed with 1 M NaOH solution (2 × 20 mL) and water (2 × 20 mL). The aqueous layers were combined and neutralized to pH = 7 with concentrated HCl on ice, then extracted using diethyl ether.

The organic phase was dried over Na_2SO_4 and concentrated to give the desired product as a yellowish or white foam.

Characterization of protected amino acids

N-4-Monomethoxytritylvaline (**MMTr-Val-OH**), 45% yield.
^{1}H NMR (400 MHz, d_6-DMSO) δ = 7.40 (4H, m, MMTr), 7.25 (6H, m, MMTr), 7.17 (2H, m, MMTr), 6.82 (2H, d, J = 8.7 Hz, MMTr), 3.71 (3H, s, OC***H*₃**), 2.94 (1H, d, J = 3.9 Hz, NHC***H***COOH), 1.68 (1H, m, C***H***$(CH_3)_2$), 0.82 (6H, t, J = 7.5 Hz, CH(C***H*₃**)$_2$).
^{13}C NMR (100 MHz, d_6-DMSO) δ = 175.0 (***C***OOH), 157.4 (C4'), 147.0, 146.7 (2×C1), 138.2 (C1'), 129.9 (2×C4), 128.4 (2×C3, 2×C5), 127.6 (2×C2, 2×C5), 126.1 (C2', C6'), 112.9 (C3', C5'), 70.2 (***C***Ph), 60.7 (NH***C***HCOOH), 54.9 (O***C***H_3), 32.9 (***C***H$(CH_3)_2$), 19.9 (CH(***C***$H_3)_2$), 17.6 (CH(***C***$H_3)_2$).
Mass: $C_{25}H_{26}NO_3^-$ [M-H$^+$]: calc. 388.2, found 388.1.

N-Tritylleucine (**Trt-Leu-OH**), 47% yield.
^{1}H NMR (400 MHz, $CDCl_3$) δ = 7.37 (6H, m, Trt), 7.15 (6H, m, Trt), 7.07 (3H, m, Trt), 3.20 (1H, t, J = 7.2 Hz, NHC***H***COOH), 1.61 (1H, m, (***C***H$(CH_3)_2$), 1.40 (2H, t, J = 7.1 Hz, CHC***H*₂**)， 0.79 (6H, dd, J = 6.5, 21.8 Hz, CH(***C***$H_3)_2$).
^{13}C NMR (100 MHz, $CDCl_3$) δ = 179.9 (***C***OOH), 145.9 (3×C1), 128.9 (3×C3, 3×C5), 127.8 (3×C2, 3×C6), 126.5 (3×C4), 71.5 (***C***Ph), 55.4 (NH***C***HCOOH), 45.1 (CH***C***H_2CH), 24.7 (***C***H$(CH_3)_2$), 23.6 (CH(***C***$H_3)_2$), 22.0 (CH(***C***$H_3)_2$).
Mass: $C_{25}H_{26}NO_2^-$ [M-H$^+$]: calc. 372.2, found 372.1.

N-Tritylproline (**Trt-Pro-OH**), 52% yield.
^{1}H NMR (400 MHz, $CDCl_3$) δ = 7.43 (6H, m, Trt), 7.23 (6H, m, Trt), 7.17 (3H, m, Trt), 4.04 (1H, dd, J = 2.5, 9.3 Hz, NC***H***COOH), 3.41 (1H, m, NC***H***$_2$), 3.00 (1H, m, NC***H***$_2$), 1.81 (1H, m, C***H***$_2$CH), 1.51 (1H, m, C***H***$_2$CH), 1.10 (1H, m, NCH_2C***H***$_2$), 0.84 (1H, m, NCH_2C***H***$_2$).
^{13}C NMR (100 MHz, $CDCl_3$) δ = 175.9 (***C***OOH), 142.9 (3×C1), 129.1 (3×C3, 3×C5), 128.2 (3×C2, 3×C6), 127.2 (3×C4), 78.8 (***C***Ph), 65.2 (N***C***HCOOH), 50.9 (N***C***H_2), 30.5 (***C***H_2CH), 24.2 (N***C***H_2CH_2).
Mass: $C_{24}H_{22}NO_2^-$ [M-H$^+$]: calc. 356.2, found 356.1.

Chemical synthesis of oligoribonucleotide:amino acid mixed anhydrides. A mixture of protected amino acids, i.e. MMTr-Val-OH (50 eq.), Trt-Leu-OH (50 eq.), Trt-Pro-OH (20 eq.), 1-ethyl-3-(3-dimethylaminopropyl)carbodiimide hydrochloride (EDC, 50 eq.) and 5-mer donor oligoribonucleotide (1 eq., 4-8 mg) were dissolved in a mixture of pyridine (600 µL) and formamide (300 µL). The resulting solution was stirred in a 0°C cold room for 2 h (proline), or 24 h (valine, leucine). Then the solution was centrifuged, and the clarified solution was transferred to a pre-cooled acetone solution of $NaClO_4$ (50 mM, 4.0 mL), and maintained at -20°C for 10 min. The product was collected as a pellet by centrifugation and was briefly dried under vacuum to remove the remaining acetone and pyridine. The white solid (ca. 4-8 mg) was dissolved in formic acid aq. (50%, 200 µL) and placed on ice for 3 min (valine, leucine, proline). Then, a pre-cooled acetone solution of $NaClO_4$ (50 mM, 4.0 mL) was added to the solution. The product was again collected as a pellet by centrifugation, briefly dried under vacuum, and immediately stored dry at -70°C for future use. The oligoribo-nucleotide:amino acid mixed anhydride pellet is stable at -70°C for at least 2 months. The purity was checked using analytical HPLC. The yields for mixed anhdrides donor RNA were >85% for Gly and L-/D-Ala, ~75% for L-Pro, ~60% for L-Leu, and ~30% for L-Val. The mass of product was characterized using LCMS by dissolving a tiny amount of the mixed anhydride in 0.2% formic acid aq. The product was characterized using ^{31}P NMR by dissolving 4 mg of the mixed anhydride in 0.2% formic acid aq. (D_2O/H_2O, 1/9). Once dissolved, the oligoribonucleotide mixed anhydrides should be stored in liquid nitrogen or at -70°C and used within one month. Ala and Gly mixed anhydrides were prepared as previously published. **^{31}P NMR** (162 MHz, D_2O) = 0.2 (5'-***P***O_3H), -1.2 – -0.5 (O***P***O_3), -7.9 (5'-***P***O_3CO).

Aminoacyl-transfer from a mixed anhydride donor to the 3'-terminus of an acceptor overhang. In a typical reaction, 5-mer oligoribonucleotide:amino acid mixed anhydride donor (6 mM in pH = 5.0, 50 mM TEAA buffer, 3 µL) was added to a solution (180 µL, 100 µM) of 10-mer oligoribonucleotide acceptor in HEPES buffer (50 mM, pH = 6.8), NaCl aq. (100 mM), $MgCl_2$ aq. (5 mM). The HPLC autosampler was set to 10°C unless otherwise noted, and samples (8 µL) were injected for analysis every 20 min. Each reaction was monitored 14-20 times. HPLC was monitored at 260 nm, with a flow rate of 1 mL/min. LC solvents: A, 10 mM triethylammonium acetate aq. (2.77 mL triethylamine and 1.50 mL acetate acid in 2.0 L ddH_2O), pH = 5.1, and B, acetonitrile. Column compartment temperature was set to 30°C. The samples were kept in the autosampler and analyzed again after 72 h to give a baseline.

Calculation of the corrected yields. Because of the incomplete coupling chemistry and/or background hydrolysis of the donor oligoribonucleotide:amino acid mixed anhydride, the yields of aminoacyl-transfer product directly determined by integration of HPLC peaks did not correspond to the yields that would have been determined had the mixed anhydride donor strand been pure. From the amount of the 5'-phosphorylated donor strand at the initial time point and the composite decay curve of the peak for the mixed anhydride thereof (by hydrolysis and transfer), it was possible to use nonlinear regression analysis to infer the relative amounts of these two materials at time zero. The yield of the aminoacyl-transfer product observed by HPLC could then be scaled to a corrected yield based on the calculated amount of mixed anhydride at time zero.

HPLC quantification and kinetic regression analysis. The peak areas of the mixed anhydride donor strand, hydrolyzed mixed anhydride, acceptor strand and diol ester product were determined at different time points. The half-life and the inferred percentage of mixed anhydride at time zero were calculated by nonlinear regression using a one-phase exponential decay, e.g., $ln(P_{mixed\ anhydride}) = kt + b$, where *t* refers to the time. Nonlinear regression using a two-phase exponential association decay, e.g., $Yield = A\,(e^{-k_1 t} - e^{-k_2 t})$ was used to calculate $k_{transfer}$ (k_2) and $k_{hydrolysis}$ (k_1). The half-life of the diol ester was calculated by $t_{1/2} = 0.69 / k_1$. The baseline, determined by the HPLC trace after at least three half-lives, was deducted from the peak areas of transfer products before the regression analysis. The time at which the concentration of the diol ester was maximum was calculated as $t_{max} = \frac{1}{(k_2 - k_1) ln\,(k_2/k_1)}$. The corrected yield was the peak yield from the nonlinear regression, e.g., $Yield_{calc.} = A\left(e^{-k_1 t_{peak}} - e^{-k_2 t_{peak}}\right)$, over the percentage of mixed anhydride at time zero. Data were processed using GraphPad Prism.

Preparation of RNA aminoacyl-transfer library. Oligoribonucleotides **Lib-L5**, **Lib-S3**, and oligodeoxyribonucleotide blocker were ordered from *Integrated DNA Technology* and purified by HPLC. **Lib-L5** and **Lib-S3** were labelled with Cyanine 5 at the 5'-terminus. DNA blocker was modified with a C3 spacer, i.e., $CH_2CH_2CH_2OH$, at the 3'-terminus. Mixed anhydrides of the donor strand **SM-1** or **Lib-S3-D** were freshly prepared as described above and stored in TEAA buffer (50 mM, pH = 5.0) in liquid nitrogen. **Lib-S3-D** mixed anhydrides were used without any purification (See Fig. S2), and were not characterized by LC-MS or ^{31}P-NMR due to the limited amount of material. **Lib-L5** or **Lib-S3** (1 mM, 1 µL), blocker (1 mM, 1 µL), HEPES buffer (500 mM, pH = 6.8, 2 µL), NaCl aq. (1000 mM, 2 µL), $MgCl_2$ aq. (50 mM, 2 µL) were mixed, heated to 95°C for 4 min then slowly cooled down to 10°C in 1 h. To the mixture, donor strand mixed anhydride **SM-1-Aa** or **Lib-S3-D-Aa** (1 mM, 2 µL) and ddH_2O were added to the final volume of 20 µL. The transfer reactions were carried out at 10°C. Transfer reactions with L-Ala, D-Ala, Gly, and L-Pro were quenched with 0.4% HCl (1 µL) after 1 h. Transfer reaction with L-Leu was quenched after 2 h; reaction with L-Val was quenched after 3 h.

Free diol oxidation. To the resulting reaction mixture (21 µL), sodium acetate aq. (50 mM, pH = 4.5, 8 µL), sodium periodate (200 mM, 5 µL), and nuclease-free water (6 µL) were added. The mixture was incubated for 40 sec at room temperature to oxidize the free 2',3'-diol of the unreacted acceptor strands before quenching by addition of ethylene glycol (2 µL) followed by thorough mixing. After further incubation for 1 min at room temperature, the reaction was adjusted to pH = 12 with NaOH aq. (1 M). Hydrolysis of the 2'-/3'-acyl ester was allowed to proceed for 30 sec and the mixture was then adjusted back to pH = 7 with HCl (1 M). RNA-grade glycogen (20 µg/µL, 2 µL) and sodium acetate (3 M, pH = 5.2, 9 µL) were then added with mixing, followed by isopropanol (93 µL). The mixture was centrifuged at 16,000 g for 20 min at 4°C. The supernatant was removed and ethanol aq. (75%, 500 µL) was added. The mixture was further centrifuged at 16,000 g for 5 min at 4°C. The supernatant was removed and the pellet was lyophilized until dry.

Ligation. Oligodeoxyribonucleotides (phosphorylated at the 5'-terminus and modified with a C3 spacer, i.e. $CH_2CH_2CH_2OH$ at the 3'-terminus) were purchased from Integrated DNA Technologies. An ImpA/$MgCl_2$ stock solution was prepared by dissolving adenosine-5'-phosphoimidazolide (ImpA, 7.1 mg) in an aqueous $MgCl_2$ solution (100 mM, 90 µL). One adaptor at a time (1 mM, 60 µL) was mixed with the ImpA/$MgCl_2$ solution (60 µL) and the resultant solution was incubated at 50°C for 1.5 h. ImpA/$MgCl_2$ solution (30 µL) and water (30 µL) were added and incubation was continued at 50°C for another 1.5 h. Water (820 µL) was added to reach 1 mL final volume. The oligonucleotides were purified using two NAP-5 columns (2 × 500 µL in parallel) according to the manufacturer's instructions. The typical yield for this reaction was 50%. In a subsequent reaction, the non-adenylated oligonucleotides were digested with Lambda Exonuclease (*NEB* M0262) according to the manufacturer's instructions and using 5 µg as input. The adaptors were subsequently purified by phenol/chloroform/ isoamyl alcohol (PCI, 25/24/1) extraction and ethanol precipitation. Finally, the resulting material was dissolved in

nuclease-free water (100 ng/µL) and analyzed via PAGE supplemented with N-acryloyl-3-aminophenylboronic acid (APB). 1 µg of each sample was mixed with T4 RNA Ligase Reaction Buffer (10×,2 µL), PEG8000 (50%, 4 µL), digested adaptor (1 µL, 100 ng), T4 RNA Ligase 2 truncated KQ (*NEB* M0373, 1 µL), and nuclease-free water (12 µL) were added and the resultant solution was incubated at 5'-Deadenylase (*NEB* M0331, 1 µL) was then added and incubation continued for 30 min at 30°C before addition of Lambda Exonuclease (*NEB* M0262, 1 µL) and further incubation for 30 min at 37°C. Finally, the reaction was purified via PCI-extraction and ethanol precipitation.

Reverse transcription. The pellet from the purified ligation reaction was dissolved in 11 µL nuclease-free water and mixed with dNTP-mix (10 mM each dATP, dCTP, dGTP, dTTP, 1 µL) and RT-primer (2 µM, 1 µL). The resultant solution was incubated at 65°C for 5 min and subsequently cooled down to 4°C. SuperScript IV RT-Buffer (5×, 4 µL), nuclease-free water (1 µL), DTT (100 mM, 1 µL) and SuperScript IV reverse transcriptase (*ThermoFisher* 18090200, 1 µL) were added and the solution was then incubated at 55°C for 10 min and at 80°C for another 10 min before being cooled down to 4°C.

PCR. The whole reverse transcription reaction (20 µL) was used as the template for PCR. It was supplemented with nuclease-free water (13.5 µL), Q5 reaction buffer (5×,10 µL), forward and reverse primer (10 µM, 2.5 µL each), dNTP-mix (10 mM each, 1 µL) and Q5 High-Fidelity DNA Polymerase (*NEB* M0491, 0.5 µL). The reaction was heated to 98°C for 30 sec then subjected to four cycles of 98°C for 10 sec, 67°C for 15 sec, and 72°C for 10 sec. Final extension was carried out at 72°C for 45 sec. The reaction products were subjected to AMPure bead purification, analyzed using Bioanalyzer DNA High Sensitivity Chips and sequenced on an iSeq 100 all according to the manufacturer's instruction.

Phosphoramidate chemistry

Chemical synthesis of oligoribonucleotide:amino acid phosphoramidates.

4-(Dimethylamino)pyridine (DMAP, 6.6 mg, 0.054 mmol) and RNA (**SM-1**, 5'-pAGCGA-3', 3 mg, 1.8 µmol, or **Lib-S3-D**, 5'-pNNNGAAUC-3', 3 mg) were dissolved in H_2O/D_2O (9:1 v/v, 0.5 mL) and the pH value was adjusted to 8.0 with hydrochloric acid (5 M). To the resulting solution, 1-ethyl-3-(3-dimethylaminopropyl)carbodiimide hydrochloride (EDC, 50 mg, 0.23 mmol) was added. The reaction mixture was kept at room temperature for 2 hours, and the reaction was followed by ^{31}P-NMR spectroscopy. Afterwards, the solution was added dropwise to a cold solution of $NaClO_4$ in acetone (50 mM, 10 mL) to precipitate the oligonucleotide-5'-DMAP phosphoramidate intermediate. The precipitate was separated by centrifugation and washed with diethyl ether (10 mL), then dried *in vacuo*. A solution of amino acid (50 mg/mL) was prepared and its pH was adjusted to 8.0 by the addition of NaOH, then 0.5 mL of this solution was added to the dried precipitate and the resultant solution was incubated at room temperature for 18 hours. The reaction was monitored by ^{31}P-NMR spectroscopy or HPLC. The **Lib-S3-D** products were used without any purification (See Fig. S4). The **SM-1** phosphoramidate products were purified using analytical HPLC. LC solvents: A, 50 mM triethylammonium acetate, pH = 7.0 in water; B, acetonitrile. Column compartment temperature: 25°C. The product was stored as a solid or dissolved in basic pH solution at -32°C for future usage. Yields for two steps: 12% for Gly, 13% for L-/D-Ala, 40% for L-Pro, 17% for L-Leu, 21% for L-Val. **^{31}P NMR** (162 MHz, D_2O) = 7.1 (5'-***P***O_3N), 0.2 (5'-***P***O_3H), -1.2 – -0.5 (O***P***O_3),

Preparation of RNA phosphoramidate-ester library. A mixture (10 µL) containing the above synthesized RNA phosphoramidate (**SM-1** phosphoramidate or **Lib-S3-D** phosphoramidate 50 µM), aminoacyl acceptor RNA (**Lib-L5** or **Lib-S3**, 50 µM), DNA blocker (Blocker**,** 50 µM), NaCl (200 mM), $MgCl_2$ (50 mM), EDC (50 mM) and imidazole (10 mM) in HEPES buffer (100 mM, pH = 7.0), was incubated at room temperature for 2 hours. Over time, 0.5 µL aliquots were taken and mixed with 2 µL of loading dye (9.5 M urea, 25 mM EDTA, pH = 8.0, blue bromophenol) before being analysed using polyacrylamide gel electrophoresis.

Ligation. The above pellet was dissolved in ddH_2O. Cyanine3 labelled 36-mer ligator was purchased from *Integrated DNA Technology*. A mixture (20 µL) of the oxidized library, or the RNA library **Lib-L5** or **Lib-S3** as blank (final concentration 10 µM), ligator (final concentration 20 µM), ATP (10 mM, 2 µL), PEG 8000 (50%, 6 µL), T4 buffer (10×, 2 µL), and T4 RNA ligase 1 (*NEB* M0204, 10 U/µL, 1 µL) was incubated at 25°C for 2 h followed by inactivation at 65°C for 15 min. The mixture was cleaned up using *NEB* Monarch RNA Cleanup Kit (*NEB* T2030, 10 µg). A 6 µL eluate was obtained. The nucleic acid concentration was checked using a NanoDrop spectrophotometer and the composition was analyzed by 12% denaturing polyacrylamide gel electrophoresis.

Reverse transcription. The resulting solution (2 μL), reverse transcription primer (RT primer, 50 μM, 2 μL), dNTP (10 mM, 1μL) and nuclease-free H_2O were mixed to a final volume of 10 μL. The mixture was heated at 65°C for 5 min and then placed on ice immediately. M-MuLV buffer (10×, 2 μL), M-MuLV reverse transcriptase (*NEB* M0253, 200 U/μL, 1 μL), RNase inhibitor (*NEB* M0314, 40 U/μL, 0.2 μL) and nuclease-free H_2O were added to the mixture to a final volume of 20 μL. The cDNA synthesis reaction was incubated at 25°C for 5 min, then 37°C for 1 h, and inactivated at 80°C for 10 min. RNase H (*NEB* M0297, 5 U/μL, 0.2 μL) was added and the mixture was further incubated at 37°C for 20 min followed by inactivation at 80°C for 10 min. The mixture was cleaned up using a Monarch PCR & DNA Cleanup Kit (*NEB* T1030, 5 μg) following the oligonucleotides cleanup protocol. A 6 μL eluate was obtained and the nucleic acid concentration therein was checked using a NanoDrop spectrophotometer.

PCR. cDNA from above reverse transcription was prepared for Illumina sequencing by PCR with Fwd and Rev primers containing Illumina adapters plus indexes. The reverse transcription product (2 μL), dNTP (10 mM, 0.5 μL), Fwd and Rev primers (10 μM, 0.5 μL each), Taq buffer (10×, 2.5 μL), Taq polymerase (*NEB* M0273, 5 U/μL, 0.125 μL) and nuclease-free water were mixed to a final volume of 25 μL. PCR amplification was carried out as follows: pre-denaturation at 95℃ for 30 s, followed by denaturation at 95°C for 30 s, annealing at 55℃ for 30 s, and extension at 68℃ for 15 s, for a total of 15 cycles. The final extension was at 68℃ for 5 min. Exonuclease I (ExoI, *Thermo Scientific*, EN0581, 20 U/μL, 2 μL) was added directly after the completion of PCR. The resultant mixture was incubated at 37℃ for 20 min, the exonuclease inactivated at 85℃ for 15 min, and the products purified using a Monarch PCR & DNA Cleanup Kit (5 μg) using the PCR cleanup protocol. The volume of eluate obtained was 10 μL, a sample was analyzed by 6% denaturing polyacrylamide gel electrophoresis, and the remainder quantified using an *Invitrogen* Qubit 2.0 Fluorometer. Next Generation Sequencing was performed on an *Illumina* MiSeq system with MiSeq Reagent Kit v3 (150-cycle) according to the methods guide from the manufacturer.

Data processing. The raw FASTQ data was first filtered and quality-controlled using custom VBA scripts in Microsoft Excel. Frameshifts were corrected and reads with unidentified bases as well as PCR duplicates were excluded. Afterwards, the results were processed, analyzed and visualized using bowtie2, PEAR, and R. 'ggpairs' was used to calculate the Pearson correlation coefficient between the results of two amino acids. The correlation is expressed with a value between -1 to 1, where -1 shows negative correlation while 1 indicates positive correlation. P-value of permutation test for Pearson correlation coefficient is displayed as '***' if the p-value is < 0.001, '**' if the p-value is < 0.01; '*' if the p-value is < 0.05; '.' if the p-value is < 0.10; ''otherwise. Scores for the selectivity of L-Ala, Gly, L-Pro, L-Leu, L-Val and L-Leu/Val aminoacyl-ester formation were calculated as the ratios of the amino acid ranking (or sum of the rankings) to the sum of all the rest amino acids rankings for the same triplet sequence.

Melting temperature measurement using MicroCal VP-Capillary DSC

Differential scanning calorimetry (DSC) experiments were performed on a *General Electric* MicroCal Variable Pressure-capillary DSC. The donor strand (without mixed anhydride) and the acceptor strand (100 μM each) were dissolved in the same buffer (400 μL) as used in the transfer reactions, i.e., 50 mM HEPES pH 6.8, 100 mM NaCl, 5 mM $MgCl_2$. The sample was initially maintained at 5°C then heated to 80°C at the rate of 2°C/min before being cooled down to 5°C at the same rate. The heating/cooling cycle was repeated three times. A blank buffer was used to calibrate the DSC curve.

Structure simulation

To obtain the characteristics of the structural ensembles adopted by the aminoacyl-tRNA overhang mimics, we used the computational energy landscape framework (*1, 2*). All simulation data is available in a zenodo repository (10.5281/zenodo.7371596).

The simulations used the AMBER OL3 force field for RNA (f99 with Barcelona α/γ backbone modifications and the χ correction for RNA (*3, 4*) in implicit solvent (igb = 2). The parameters for the amino acyl groups were derived in the follows. The amino acid equivalent N-terminal residue from the ff19SB force field (*5*) was used as template. We edited this residue manually to add a phosphate group with a methyl cap. The structure was then optimised with Gaussian (*6*) and charges fitted using RESP (*7*). The library file was then created by modifying the entries for the corresponding N-terminal amino

acid form the AMBER library adding the missing information and updating the coordinates and charges. A single missing parameter, the CX-C-OS angle, was added as force modification file from GAFF. The libraries for the L-amino-acyl groups for Ala, Pro, Leu and Val, the D-amino-acyl group for Ala and the amino-acyl group for Gly are available in the online repository.

We used RNAcomposer (*8*) to obtain initial three-dimensional structures. As this tool requires a single chain, we added a UUUUU segment between the strands and enforced basepairing in the stem. The U loop was then removed, and the aaX group added. Basin-hopping global optimisation (*9*) was used to find low energy configurations, and the lowest 50 structures saved. We used short and long BH runs of 5000 and 25000 steps, respectively. The low-lying structures from both runs were used to seed our explorations of the energy landscape.

Fig. S1. Schematic representation of the library preparation workflow.

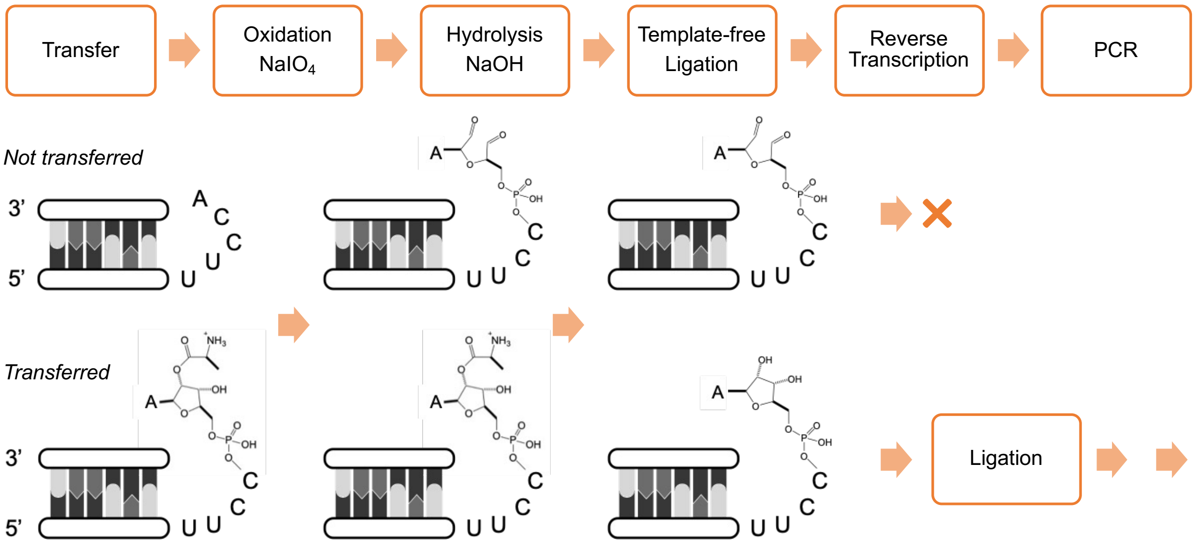

Fig. S2. Consistent patterns in the overhang. Plot of sequencing results for different penta-nucleotides in the overhang (5′-UCGCUNNNNN) to show the correlation between amino acids: (**A**) mixed anhydride chemistry; (**B**) phosphoramidate chemistry. Axes of the scatter plots are read numbers and are omitted for simplicity. Sequence logos represent the top 10% of pentanucleotides from the quality-filtered read numbers. Pearson correlation coefficients are shown in the upper right corners.

(A)

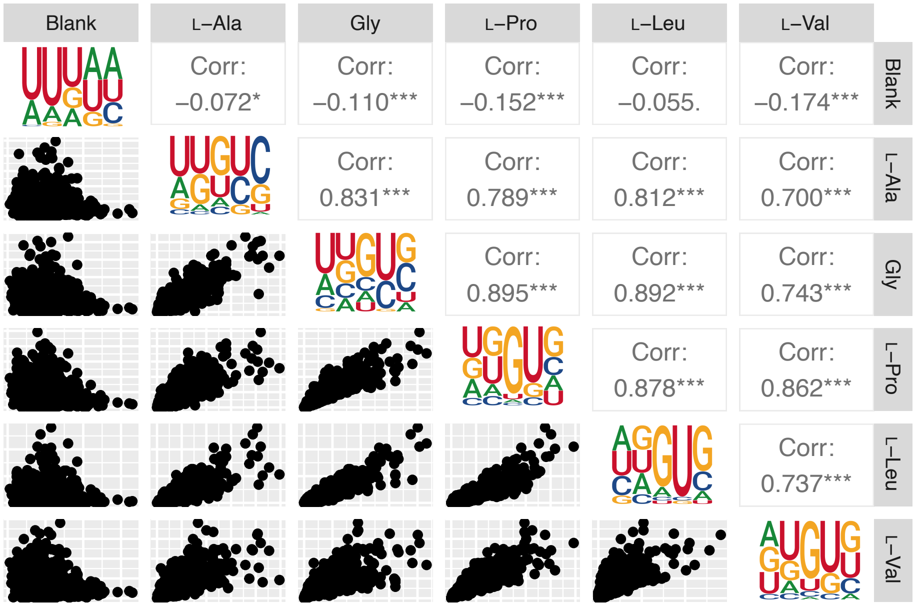

(B)

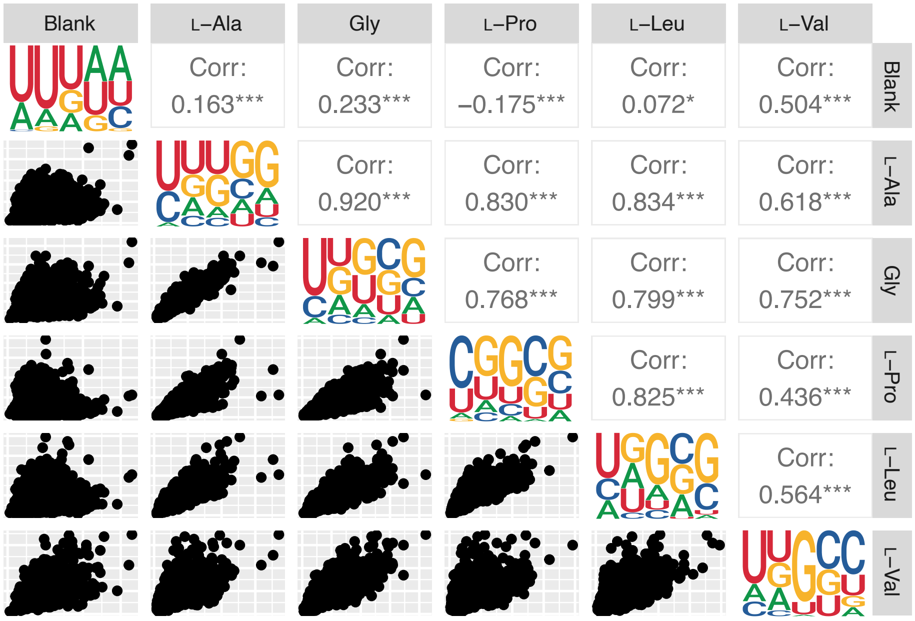

Fig. S3. Formation of RNA:amino acid mixed anhydrides with **Lib-S3-D.** HPLC conditions: Atlantis™ T3, 5 µm, 4.6 x 250 mm column; flow rate 1 mL/min; LC solvents: A, 10 mM triethylammonium acetate, pH 5.1 in water and B, acetonitrile. Gradient: 0 min (7% B), to 1 min (7% B), to 15 min (20% B), to 15.5 min (95% B). Column compartment temperature is set at 25°C. Chromatograms, in descending order, show the HPLC profiles for the unmodified library and oligonucleotide mixed anhydrides.

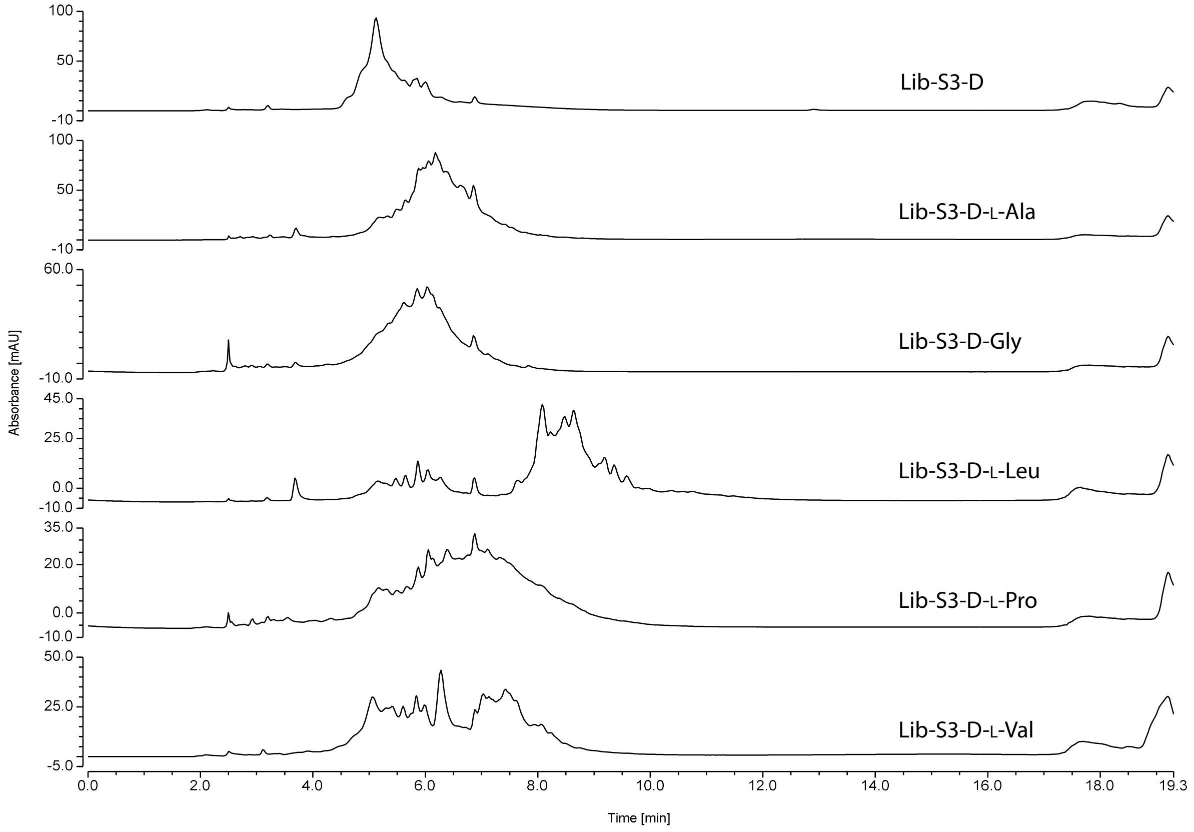

Fig. S4. Sequencing results for acceptor strand with random trinucleotide in the stem (**Lib-S3**, and **Lib-S3-D** 5'-pNNNGAAUC) using mixed anhydride chemistry. Reads for the trinucleotide CUC omitted from the glycine data to enable visualization of other sequence preferences. Sequence logo represents top 10% trinucleotides from the raw reading numbers. Pearson correlation coefficients are shown in the upper right corner.

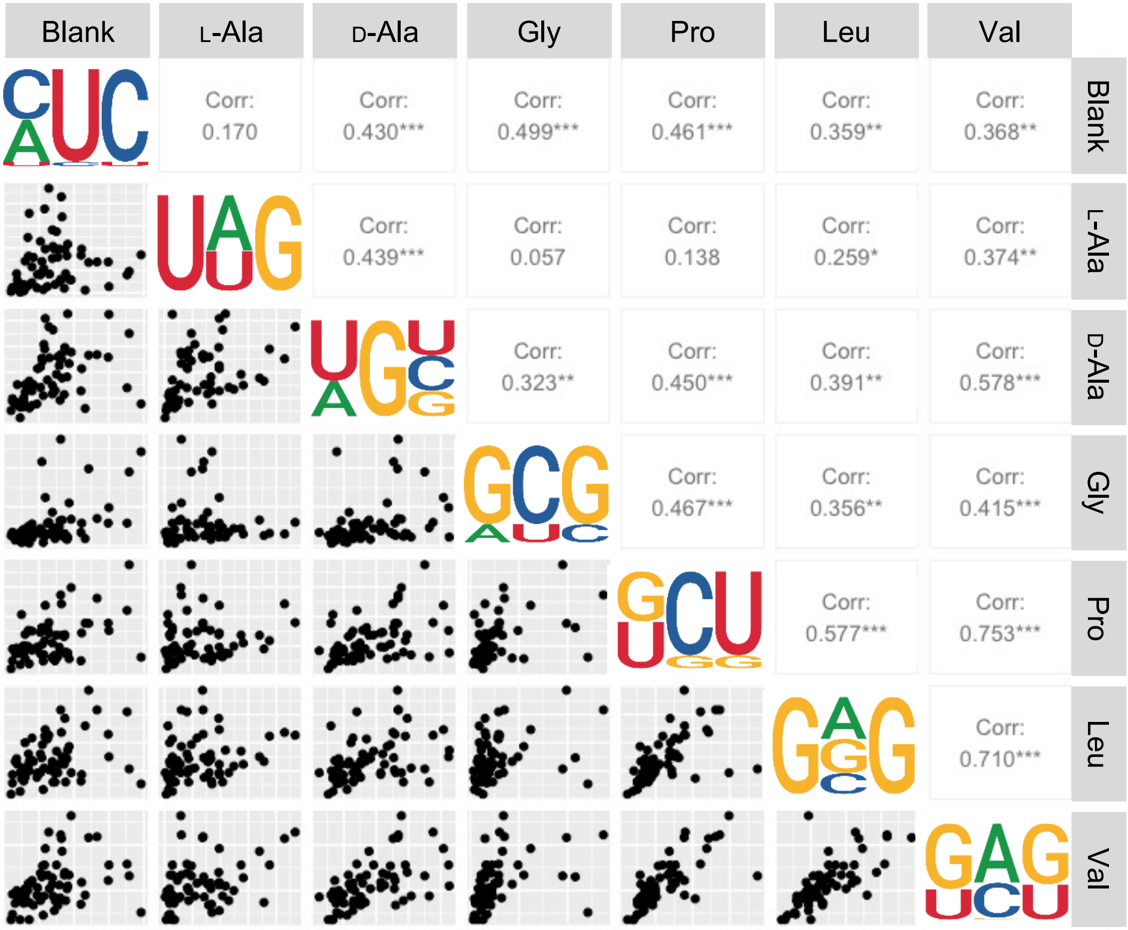

Fig. S5. Formation of RNA:amino acid phosphoramidate with **Lib-S3-D.** HPLC conditions: Atlantis™ T3, 5 µm, 4.6 x 250 mm column; flow rate 1 mL/min; LC solvents: A, 20 mM triethylammonium acetate, pH 7 in water and B, acetonitrile. Gradient: 0 min (7% B), to 1 min (7% B), to 20 min (12% B), to 22 min (16% B) and 23 min (95% B). Column compartment temperature is set at 25°C. Chromatograms, in descending order, show the HPLC profiles for the unmodified library; the library after conversion to a DMAP phosphoramidate intermediate; products of reaction of the DMAP phosphoramidate intermediate with the amino acids indicated.

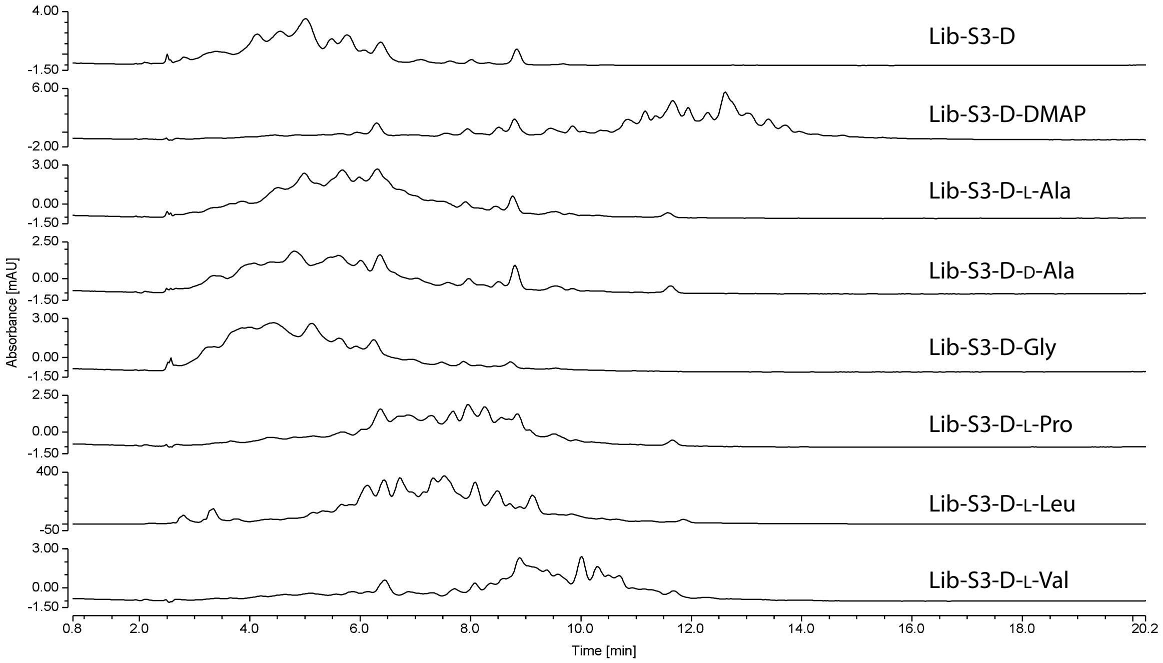

Fig. S6. Consistent patterns in the first three nucleotides of the stem using phosphor-amidate chemistry. Plot of sequencing results for different trinucleotides in the stem (5′-GAUUCNNNUUCCA) to show the correlation between amino acids. Sequence logos represent the top 10% of trinucleotides from the quality-filtered read numbers. Pearson correlation coefficients are shown in the upper right corner.

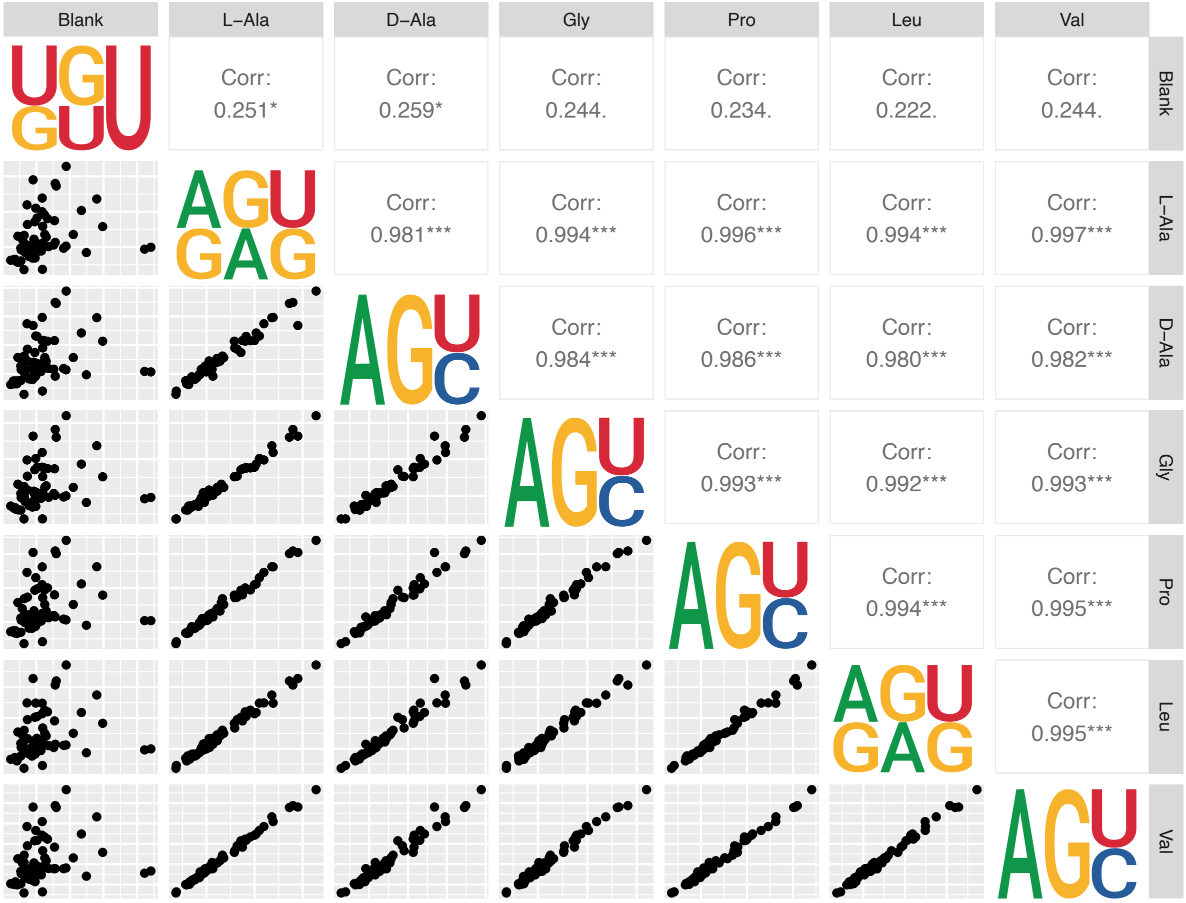

Fig. S7. DSC melting curves of 100 μM duplex **SM-11-Acc** with **SM-11** (black), **SM-16** (blue), and **SM-17** (orange). Curve in gray shows the blank using buffer, e.g. 50 mM HEPES, 100 mM NaCl, 5 mM $MgCl_2$, pH = 6.8.

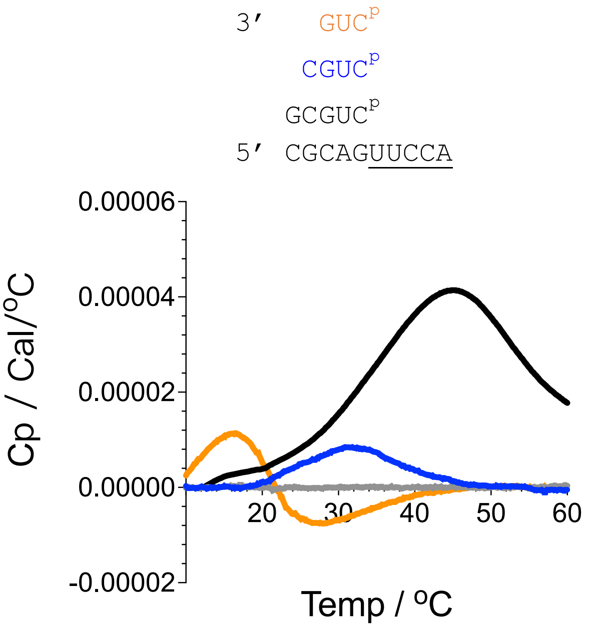

Fig. S8. Disconnectivity graphs (*10, 11*) for the free energy landscapes of L-alanyl- and L-leucyl- transfer using the sequences of donor and acceptor strands indicated. (**A**) **SM-18-Ala**; (**B**) **SM-4-Ala**; (**C**) **SM-6-Leu**; (**D**) **SM-19-Leu**. The order parameters are as follows. For the Ala sequences (**A** and **B**) the colour indicates the distances between the NH_3^+ group of the amino acid attached to the acceptor and the exocyclic N^6 in the Hoogsteen edge of adenosine in the third base pair (**SM-18-Ala**, **A**) or the O^6 in the guanine in the third base pair (**SM-4-Ala**, **B**). For the Leu sequences (**C** and **D**), it is the smallest distance between the bridging anhydride oxygen bnetween P and C in the phosphate linking Leu with the first nucleotide, and the Hoogsteen edge in the second base pair (O^6 in guanine for **SM-6-Leu**, **C**, and exocyclic N^4 in cytosine for **SM-19-Leu**, **D**). Green and blue indicate distances beyond 10 Å, while red highlights closer proximity. For both **SM-4-Ala** and **SM-19-Leu** (**B** and **D**), i.e. the sequences with lower observed yields, such contacts are formed in the lowest energy structures, but they are absent in **SM-18-Ala** and **SM-6-Leu** (**A** and **C**). The contacts make a close approach of the overhang to the amino acyl group geometrically challenging, and, as a result, we expect an activation barrier for the transfer, as these contacts need to be lost first. This effect likely lowers the yield, but the contacts are not strong enough to completely suppress the reaction.

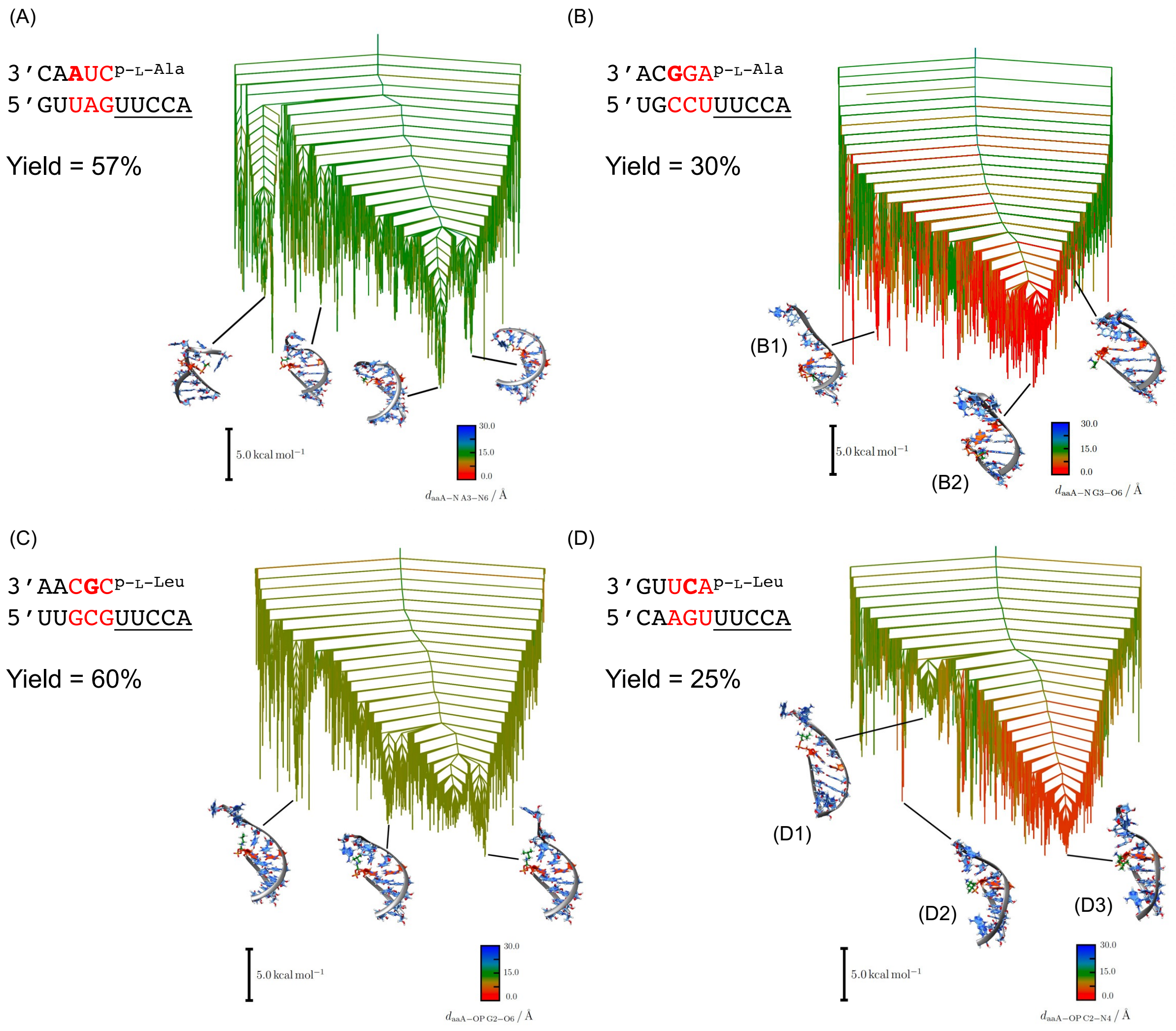

Fig. S9. Representative structures for L-Leu and L-Val selected from the energy landscape databases for two sequence combinations with different preferences. Kinetic curves shown in **Fig. 4A** (**A**-**C**) and **4B** (**D**-**F**), kinetic data presented in **Tables S18** (**A**-**C**) and **S19** (**D**-**F**). In **SM-11-Leu** (**A** and **B**) and **SM-11-Val** (**C**), the Leu and Val amino acyl groups form some interactions, but they are effectively free to move, and the transfer reaction should be possible. For **SM-13-Leu** (**D**) this picture is unchanged, and we therefore find no effect that would impede the transfer reaction. However, in **SM-13-Val**, i.e. the same sequence but with Val, we see significant changes. Stronger interactions are formed, which either result in interactions with and stacking of the overhang (**E**) or the AU base pair on top of the stem is lost, and strong interactions are formed (**F**). Both of these changes result in a barrier to the transfer explaining why the Val transfer has a significantly lower yield than the Leu transfer. Given the position of the side chain of Leu in the structures, and the strong charge interactions in RNA, wespeculate that the behaviour is based on the difference in hydrophobicity. The amino moiety is highlighted in green and the top base pair in orange. The images were created with Chimera (*12*).

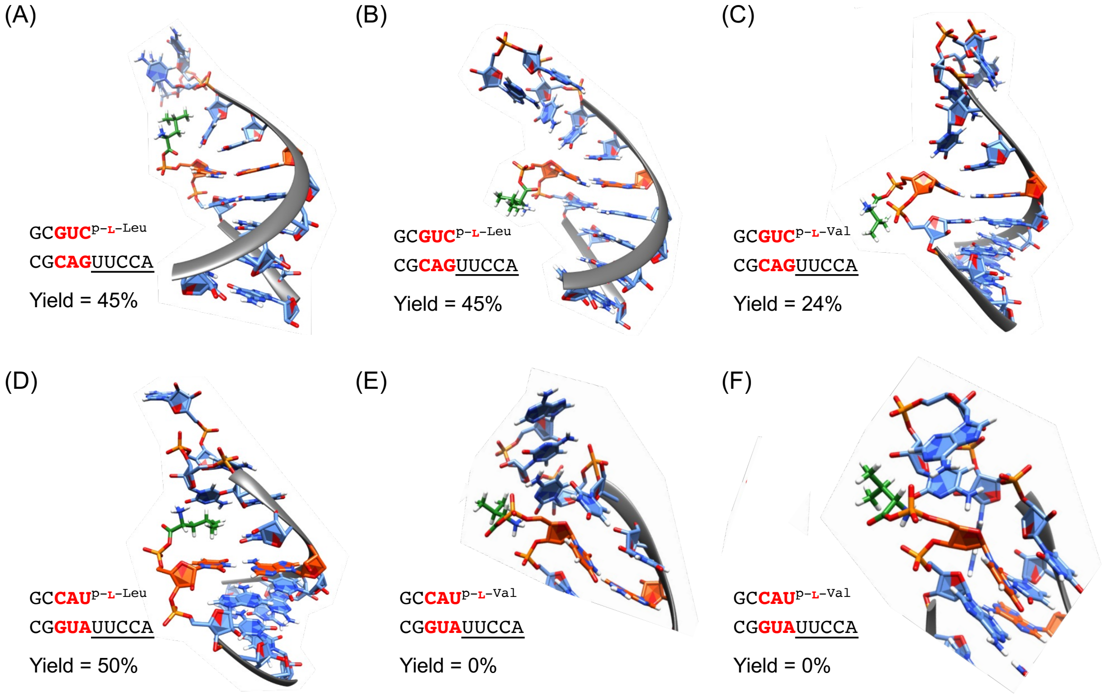

Fig. S10. ^{1}H- and ^{13}C-NMR for *N*-4-monomethoxytritylvaline (contains triethylamine)

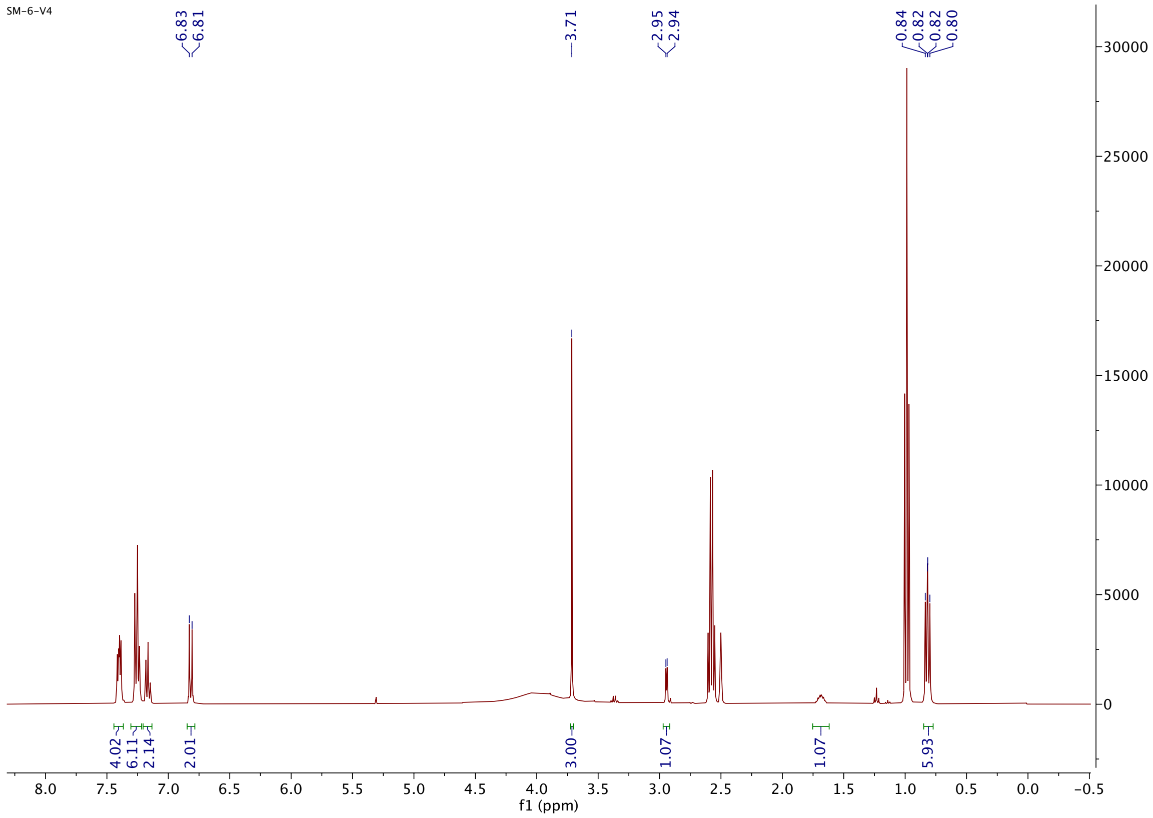

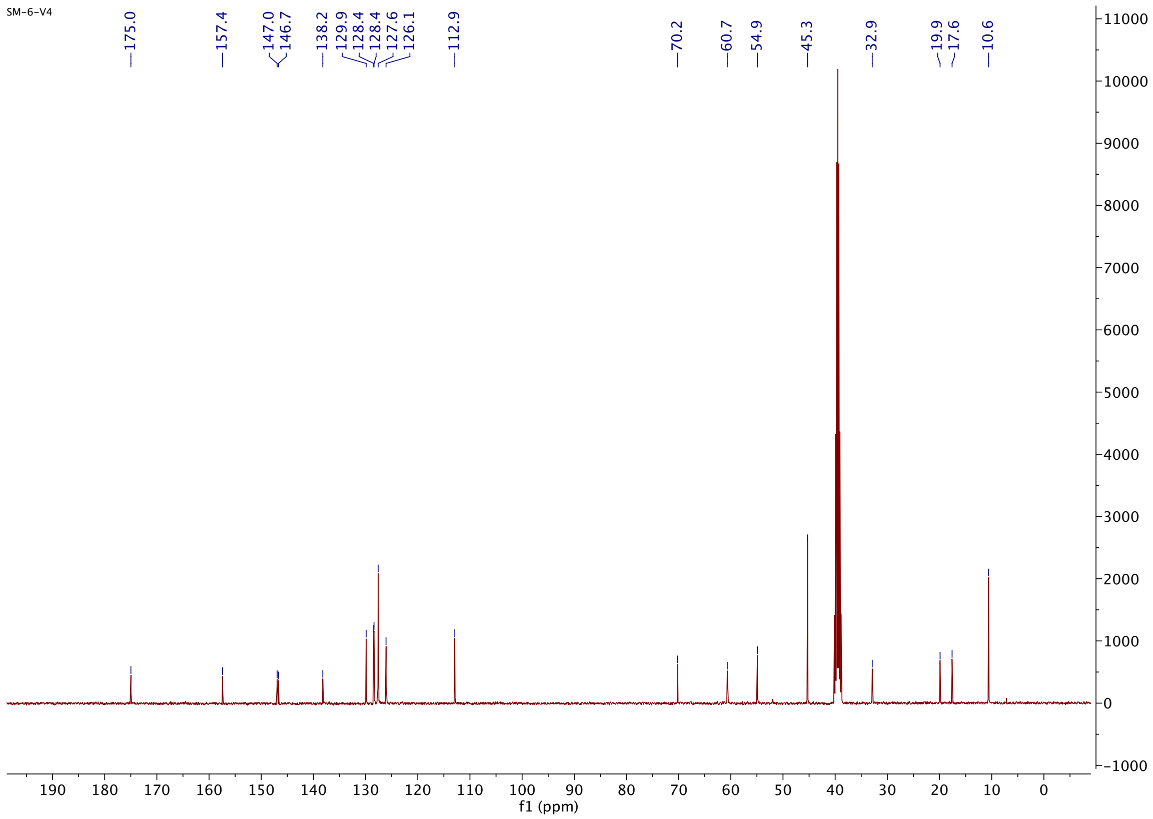

Fig. S11. ^{1}H- and ^{13}C-NMR for *N*-tritylleucine

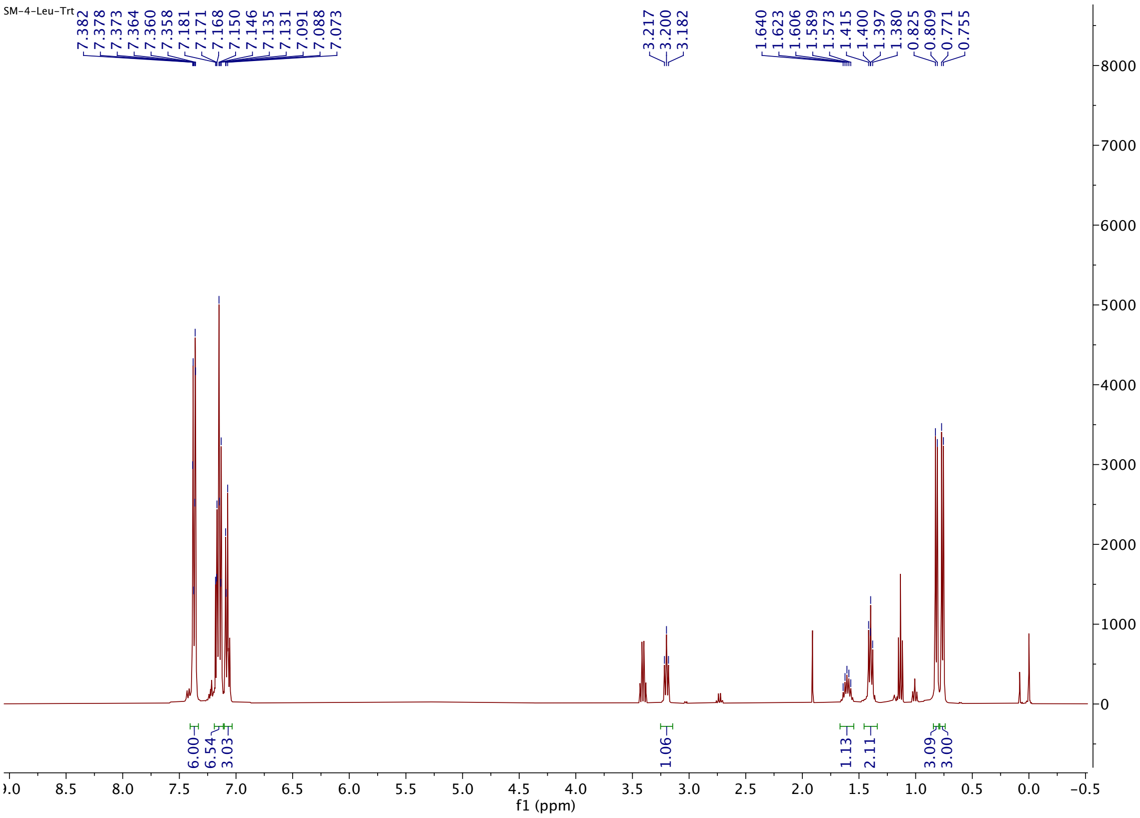

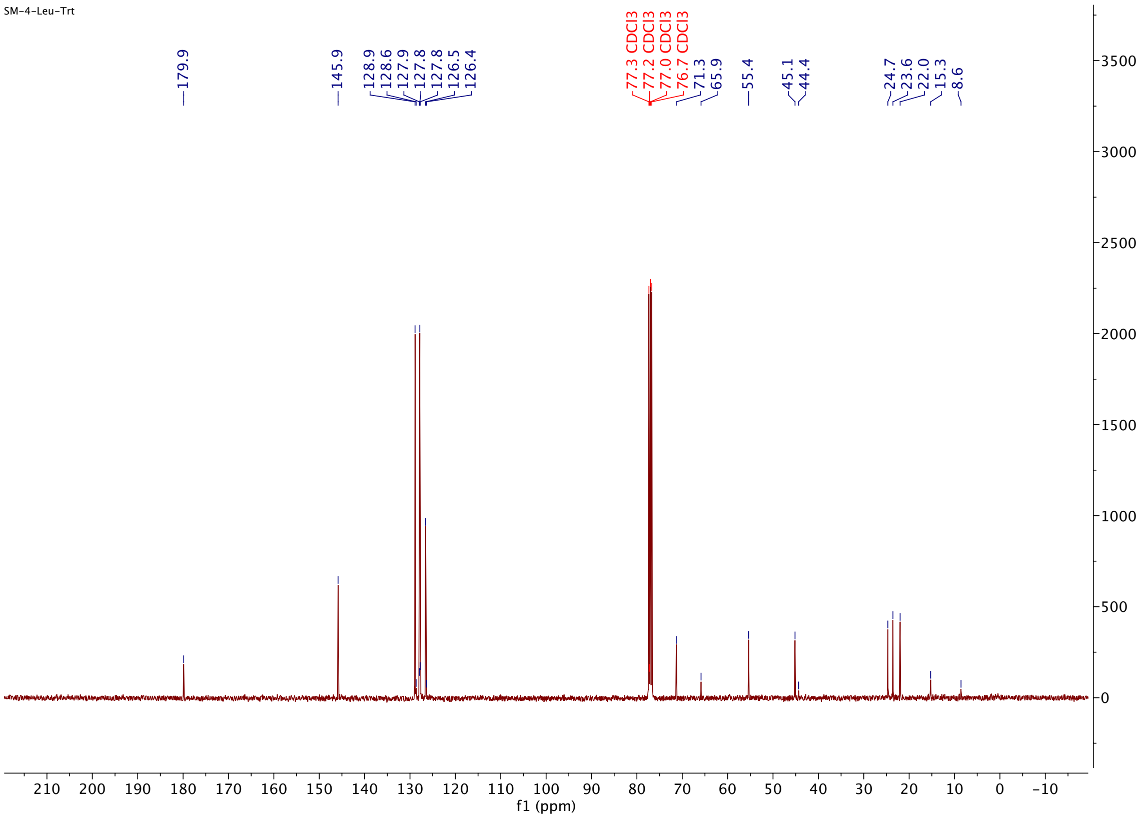

Fig. S12. ^{1}H- and ^{13}C-NMR for *N*-tritylproline

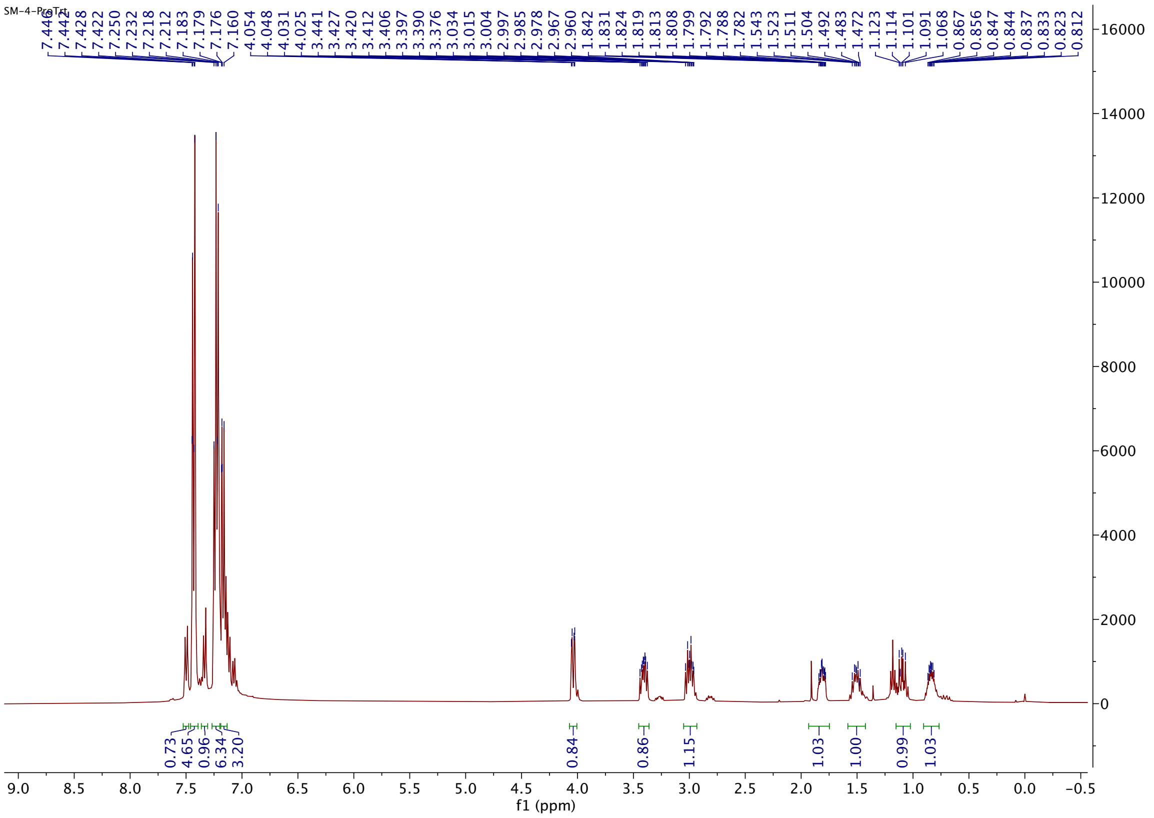

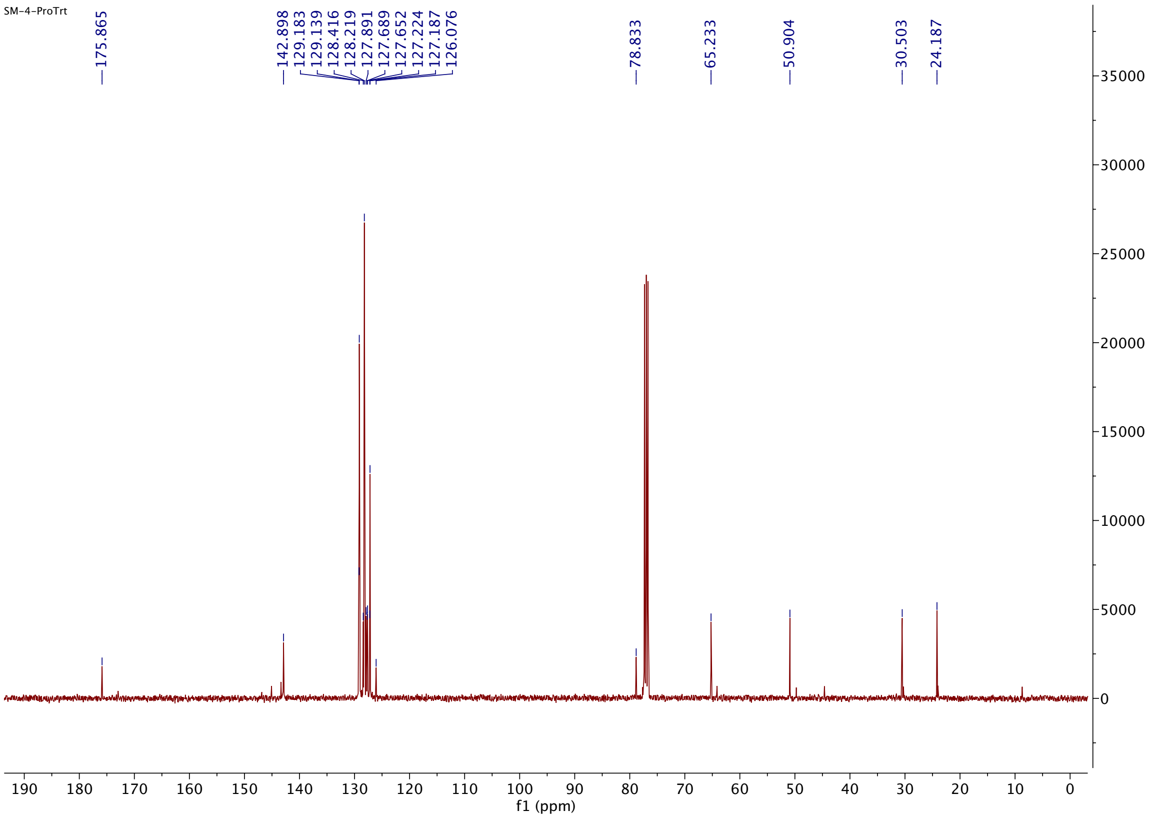

Fig. S13. Valine transfer in a tRNA acceptor stem SM-1. Duplex sequence:

5'UCGCUUUCCA

3'AGCGAp-L-Val;

Transfer was monitored using HPLC with 260 nm UV detection. The solution was incubated at 10°C and aliquots of 8 μL were injected into an HPLC at different time points. Peaks for the donor, the donor mixed anhydride and acceptor strands are indicated. The peak presumed to be due to the diol ester transfer product is highlighted by the dashed box. Conditions: both oligos (100 μM), NaCl (100 mM), $MgCl_2$ (5 mM), HEPES (50 mM, pH 6.8).

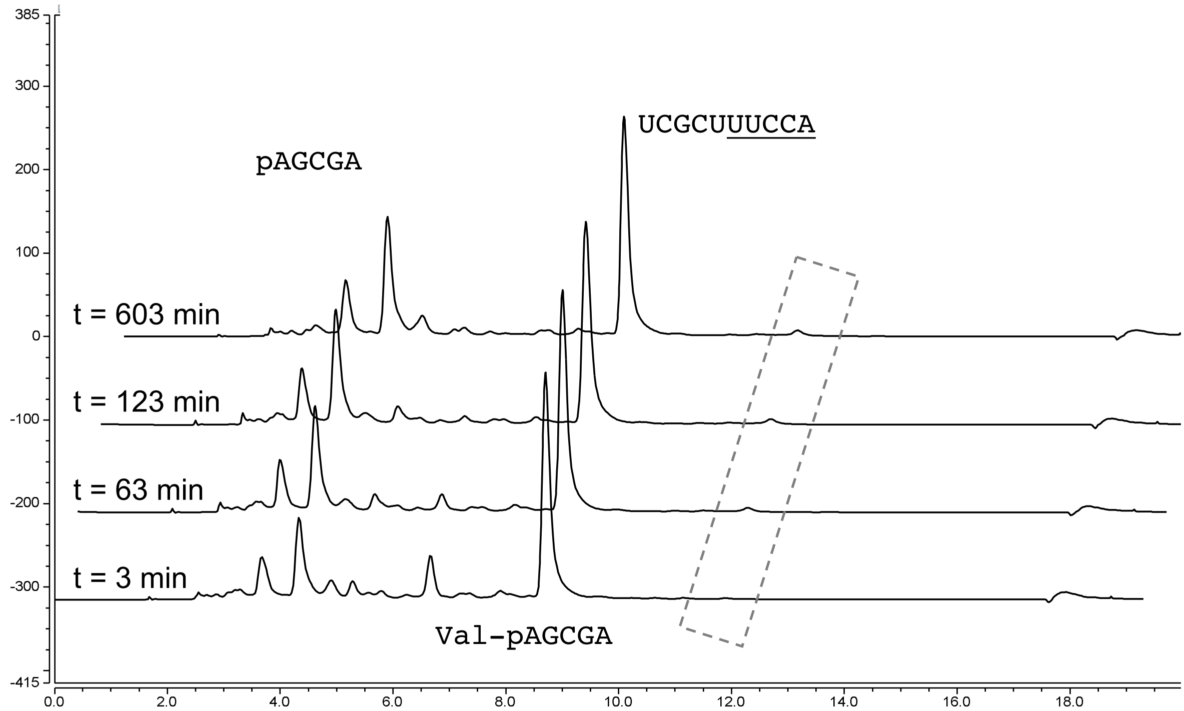

Fig. S14. Leucine transfer in a tRNA acceptor stem SM-1. Duplex sequence:

5'UCGCUUUCCA

3'AGCGAp-L-Leu;

Transfer was monitored using HPLC with 260 nm UV detection. The solution was incubated at 10°C and aliquots of 8 µL were injected into an HPLC at different time points. Peaks for the donor, the donor mixed anhydride and acceptor strands are indicated. The peak presumed to be due to the diol ester transfer product is highlighted by the dashed box. Conditions: both oligos (100 µM), NaCl (100 mM), $MgCl_2$ (5 mM), HEPES (50 mM, pH 6.8).

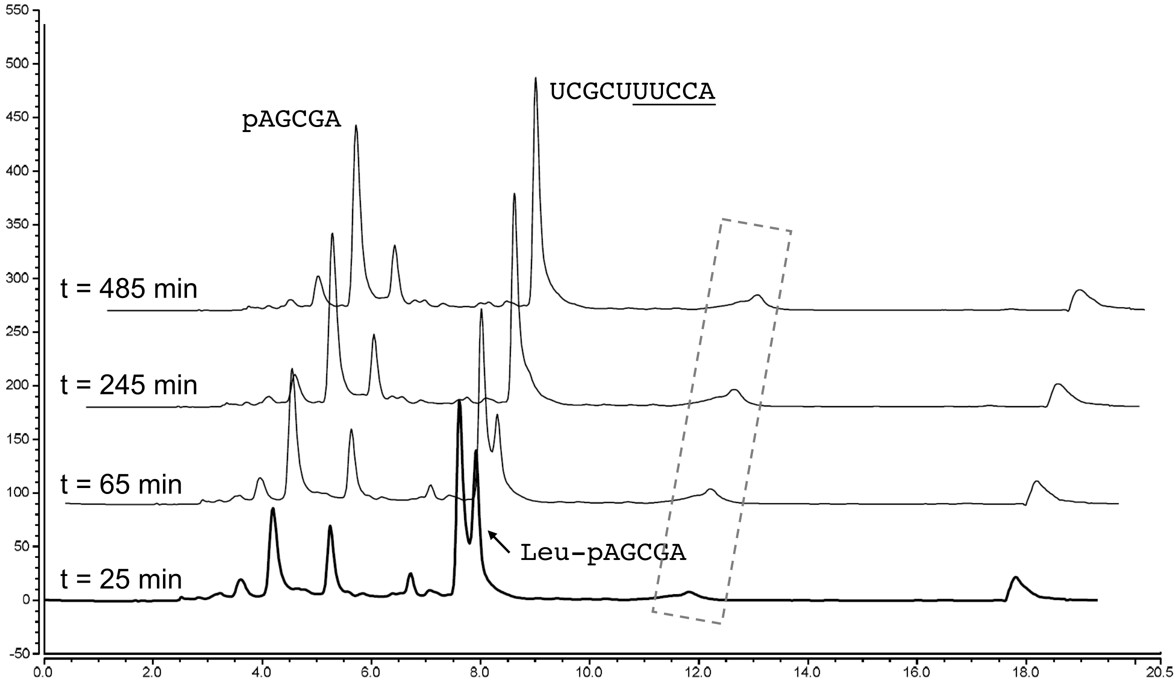

Fig. S15. Proline transfer in a tRNA acceptor stem SM-1. Duplex sequence:

5'UCGCUUUCCA

3'AGCGAp-L-Pro;

Transfer was monitored using HPLC with 260 nm UV detection. The solution was incubated at 10°C and aliquots of 8 µL were injected into an HPLC at different time points. Peaks for the donor, the donor mixed anhydride and acceptor strands are indicated. The peak presumed to be due to the diol ester transfer product is highlighted by the dashed box. Conditions: both oligos (100 µM), NaCl (100 mM), $MgCl_2$ (5 mM), HEPES (50 mM, pH 6.8).

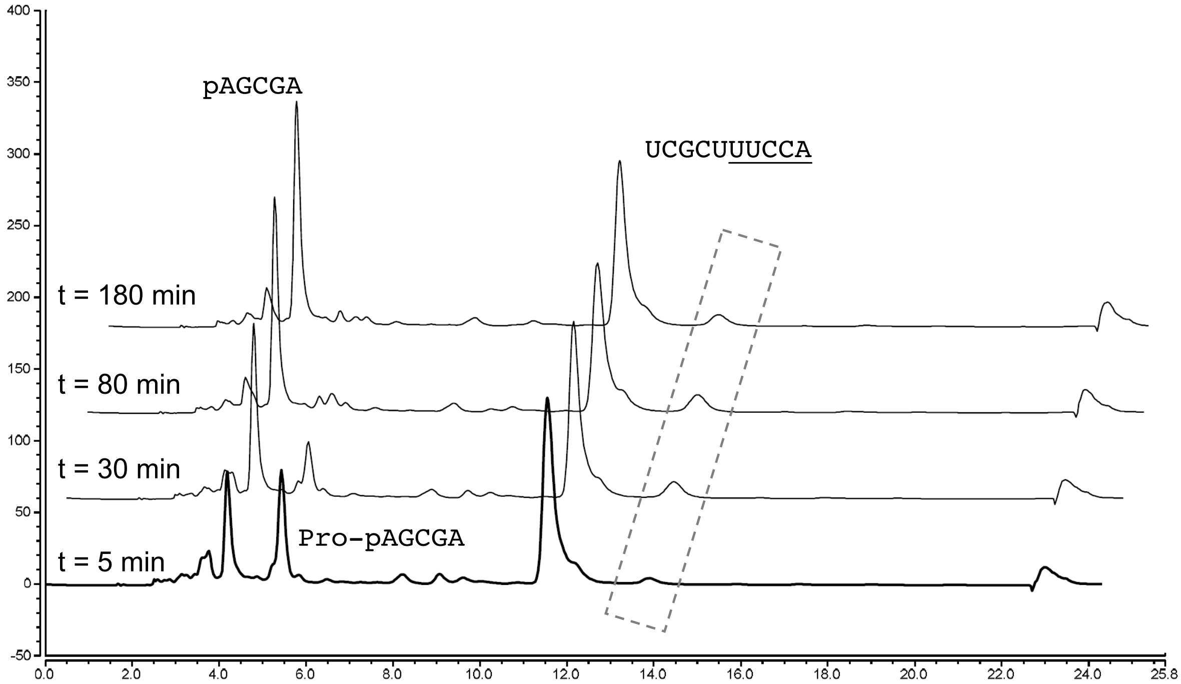

Table S1. Sequence of the donor and acceptor strands used in the mixed anhydride chemistry. Found, observed (raw) mass number from LCMS in negative ion mode; Expt., the experimental mass number calculated from the observed values, i.e. (Found + 1) × charges, 2 charges for 5-mer Aa donors and 3 charges for 10-mer acceptors; Calc., mass number in theory; Acc, acceptor.

Stem		Sequence	mer	Calc.	Expt.	Found
**SM-1**	SM-1-Acc	UCGCU UUCCA	10	3056.4	3056.7	1017.9
	Acc-1A	UCGCU AGCCA	10	3118.5	3118.8	1038.6
	Acc-1C	UCGCU CGCCA	10	3094.5	3094.5	1030.5
	Acc-1G	UCGCU GGCCA	10	3134.5	3134.4	1043.8
	Acc-2A	UCGCU UACCA	10	3079.4	3079.2	1025.4
	Acc-2C	UCGCU UCCCA	10	3055.4	3055.5	1017.5
	Acc-2U	UCGCU UUCCA	10	3056.4	3056.7	1017.9
	Acc-3A	UCGCU UGACA	10	3119.4	3119.4	1038.8
	Acc-3G	UCGCU UGGCA	10	3135.3	3135.3	1044.1
	Acc-3U	UCGCU UGUCA	10	3096.4	3096.3	1031.1
	Acc-UA	UCGCU UUACA	10	3080.4	3080.1	1025.7
	Acc-UG	UCGCU UUGCA	10	3096.4	3096.3	1031.1
	Acc-UU	UCGCU UUUCA	10	3057.4	3057.0	1018.0
	Acc-4A	UCGCU UGCAA	10	3119.5	3119.4	1038.8
	Acc-4G	UCGCU UGCGA	10	3135.5	3135.6	1044.2
	Acc-4U	UCGCU UGCUA	10	3096.4	3096.9	1031.3
	Acc-5C	UCGCU UGCCC	10	3071.4	3071.7	1022.9
	Acc-5G	UCGCU UGCCG	10	3111.4	3111.3	1036.1
	Acc-5U	UCGCU UGCCU	10	3072.4	3072.6	1023.2
	SM-1	pAGCGA	5	1671.3	1671.2	834.6
	SM-1-Gly	Gly-pAGCGA	5	1728.3	1728.0	863.0
	SM-1-Ala	Ala-pAGCGA	5	1742.3	1742.0	870.0
	SM-1-Val	Val-pAGCGA	5	1770.4	1768.8	883.4
	SM-1-Leu	Leu-pAGCGA	5	1784.4	1784.0	891.0
	SM-1-Pro	Pro-pAGCGA	5	1768.4	1768.2	883.1
**SM-2**	SM-2-Acc	UUGCU UUCCA	10	3057.4	3057.3	1018.1
	SM-2	pAGCAA	5	1655.3	1655.0	826.5
	SM-2-Gly	Gly-pAGCAA	5	1712.3	1712.0	855.0
	SM-2-Ala	Ala-pAGCAA	5	1726.3	1726.0	862.0
	SM-2-Val	Val-pAGCAA	5	1754.4	1754.0	876.0
	SM-2-Leu	Leu-pAGCAA	5	1768.4	1768.0	883.0
	SM-2-Pro	Pro-pAGCAA	5	1752.4	1752.0	875.0
**SM-3**	SM-3-Acc	UCACU UUCCA	10	3040.4	3040.2	1012.4
	SM-3	pAGUGA	5	1672.2	1672.0	835.0
	SM-3-Gly	Gly-pAGUGA	5	1729.2	1729.0	863.5
	SM-3-Ala	Ala-pAGUGA	5	1743.2	1743.0	870.5
	SM-3-Val	Val-pAGUGA	5	1771.4	1771.0	884.5
	SM-3-Leu	Leu-pAGUGA	5	1785.3	1785.0	891.5

	SM-3-Pro	Pro-pAGUGA	5	1769.4	1769.0	883.5
**SM-4**	SM-4-Acc	UGCCU UUCCA	10	3056.4	3056.4	1017.8
	SM-4	pAGGCA	5	1671.3	1671.0	834.5
	SM-4-Gly	Gly-pAGGCA	5	1728.3	1727.8	862.9
	SM-4-Ala	Ala-pAGGCA	5	1742.3	1741.8	869.9
	SM-4-Val	Val-pAGGCA	5	1770.4	1770.0	884.0
	SM-4-Leu	Leu-pAGGCA	5	1784.4	1784.0	891.0
	SM-4-Pro	Pro-pAGGCA	5	1768.4	1768.0	883.0
**SM-5**	SM-5-Acc	UCGUU UUCCA	10	3057.4	3057.3	1018.1
	SM-5	pAACGA	5	1655.3	1655.0	826.5
	SM-5-Gly	Gly-pAACGA	5	1712.3	1711.8	854.9
	SM-5-Ala	Ala-pAACGA	5	1726.3	1726.0	862.0
	SM-5-Val	Val-pAACGA	5	1754.4	1754.0	876.0
	SM-5-Leu	Leu-pAACGA	5	1768.4	1768.0	883.0
	SM-5-Pro	Pro-pAACGA	5	1752.4	1751.8	874.9
**SM-6**	SM-6-Acc	UUGCG UUCCA	10	3096.4	3096.3	1031.1
	SM-6	pCGCAA	5	1631.2	1631.0	814.5
	SM-6-Gly	Gly-pCGCAA	5	1688.2	1688.0	843.0
	SM-6-Ala	Ala-pCGCAA	5	1702.2	1702.0	850.0
	SM-6-Val	Val-pCGCAA	5	1730.4	1730.0	864.0
	SM-6-Leu	Leu-pCGCAA	5	1744.3	1744.0	871.0
	SM-6-Pro	Pro-pCGCAA	5	1728.4	1728.0	863.0

Stem		Sequence	mer	Calc.	Expt.	Found
**SM-11**	SM-11-Acc	CGCAG UUCCA	10	3118.5	3118.5	1038.5
	SM-11U-Acc	CGUAG UUCCA	10	3119.5	3120.0	1039.0
	SM-11	pCUGCG	5	1624.2	1624.0	811.0
	SM-11-Gly	pCUGCG	5	1681.2	1681.0	839.5
	SM-11-L-Ala	pCUGCG	5	1695.2	1693.4	845.7
	SM-11-D-Ala	pCUGCG	5	1695.2	1695.0	846.5
	SM-11-Val	pCUGCG	5	1723.4	1723.0	860.5
	SM-11-Leu	pCUGCG	5	1737.3	1735.6	866.8
	SM-11-Pro	pCUGCG	5	1721.4	1720.8	859.4
**SM-12**	SM-12-Acc	CGCUC UUCCA	10	3055.4	3055.2	1017.4
	SM-12	pGAGCG	5	1687.3	1687.0	842.5
	SM-12-Gly	pGAGCG	5	1744.3	1743.8	870.9
	SM-12-L-Ala	pGAGCG	5	1758.3	1758.0	878.0
	SM-12-D-Ala	pGAGCG	5	1758.3	1758.0	878.0
	SM-12-Val	pGAGCG	5	1786.4	1786.0	892.0
	SM-12-Leu	pGAGCG	5	1800.4	1800.4	899.2
	SM-12-Pro	pGAGCG	5	1784.4	1783.8	890.9
**SM-13**	SM-13-Acc	CGGUA UUCCA	10	3119.5	3119.4	1038.8
	SM-13	pUACCG	5	1608.2	1608.0	803.0
	SM-13-Gly	pUACCG	5	1665.2	1665.2	831.6
	SM-13-L-Ala	pUACCG	5	1679.2	1679.6	838.8
	SM-13-D-Ala	pUACCG	5	1679.2	1679.0	838.5
	SM-13-Val	pUACCG	5	1707.4	1706.8	852.4
	SM-13-Leu	pUACCG	5	1721.3	1719.4	858.7
	SM-13-Pro	pUACCG	5	1705.4	1705.0	851.5
**SM-14**	SM-14-Acc	CGGAG UUCCA	10	3158.5	3158.4	1051.8
	SM-14	pCUCCG	5	1584.2	1584.0	791.0
	SM-14-Gly	pCUCCG	5	1641.2	1641.0	819.5
	SM-14-L-Ala	pCUCCG	5	1655.2	1655.2	826.6
	SM-14-D-Ala	pCUCCG	5	1655.2	1654.8	826.4
	SM-14-Val	pCUCCG	5	1683.4	1683.0	840.5
	SM-14-Leu	pCUCCG	5	1697.4	1696.8	847.4
	SM-14-Pro	pCUCCG	5	1681.3	1681.0	839.5
**SM-15**	SM-15-Acc	CGUCU UUCCA	10	3056.4	3056.4	1017.8
	SM-15	pAGACG	5	1671.3	1671.2	834.6
	SM-15-Gly	pAGACG	5	1728.3	1728.0	863.0
	SM-15-L-Ala	pAGACG	5	1742.3	1742.0	870.0
	SM-15-D-Ala	pAGACG	5	1742.3	1741.8	869.9
	SM-15-Val	pAGACG	5	1770.4	1770.4	884.2
	SM-15-Leu	pAGACG	5	1784.4	1784.0	891.0
	SM-15-Pro	pAGACG	5	1768.4	1768.2	883.1

**SM-16**	SM-16	pCUGC	4	1279.2	1279.0	638.5
	SM-16-L-Ala	pCUGC	4	1350.2	1350.0	674.0
	SM-16-Leu	pCUGC	4	1392.4	1392.0	695.0
	SM-16-Pro	pCUGC	4	1376.3	1376.0	687.0
**SM-17**	SM-17	pCUG	3	974.1	973.9	972.9
	SM-17-L-Ala	pCUG	3	1045.1	1044.9	1043.9
	SM-17-Leu	pCUG	3	1087.3	1086.9	1085.9
	SM-17-Pro	pCUG	3	1071.3	1070.9	1069.9
**SM-18**	SM-18-Acc	GUUAG UUCCA	10	3120.4	3120.3	1039.1
	SM-18	pCUAAC	5	1592.2	1592.4	795.2
	SM-18-Ala	pCUAAC	5	1663.2	1663.0	830.5
**SM-19**	SM-19-Acc	CAAGU UUCCA	10	3103.5	3103.2	1033.4
	SM-19	pACUUG	5	1609.2	1609.0	803.5
	SM-19-Leu	pACUUG	5	1722.4	1722.0	860.0

Table S2. Kinetic data of L-alanyl-transfer with varied overhangs using mixed anhydride chemistry. Aminoacyl-transfer reactions were conducted at 10°C in 50 mM HEPES, 100 mM NaCl, 5 mM $MgCl_2$, pH = 6.8 buffer with 100 μM equal equivalent of donor strand and acceptor strand, unless otherwise noted in **Fig. 3H**. UGCCA, UGACA and UGUCA shows similar transfer yield and kinetics. This is due to the overhang palindromic structure where nicked duplex transfer occurred, along with the self-folding nicked loop transfer.

	Donor	Acceptor		Observed yield	Corrected yield	Mixed anhyd. half-life /h	Diol ester half-life /h	Peak time /min	k transfer /min^{-1}	k hydrolysis /min^{-1}
		Stem	Overhang							
**SM-1**	L-Ala-pAGCGA	UCGCU	UGCCA	30%	55%	0.2	4.1	43	0.0780	0.00279
			AGCCA	0%	0%	0.8	--	--	--	--
			CGCCA	6%	9%	0.8	20	89	0.0508	0.00058
			GGCCA	0%	0%	--	--	--	--	--
			UACCA	16%	30%	0.4	12	80	0.0503	0.00095
			UCCCA	0%	0%	0.2	--	--	--	--
			UUCCA	34%	57%	0.5	10	70	0.0569	0.00116
			UGACA	23%	56%	0.2	5.0	32	0.1278	0.00229
			UGGCA	0%	0%	0.5	--	--	--	--
			UGUCA	24%	59%	0.1	1.8	21	0.1585	0.00648
			UUACA	28%	39%	0.3	6.1	52	0.0717	0.00190
			UUGCA	0%	0%	0.2	--	--	--	--
			UUUCA	35%	45%	0.2	7.5	42	0.1008	0.00153
			UGCAA	0%	0%	0.2	--	--	--	--
			UGCGA	0%	0%	0.8	--	--	--	--
			UGCUA	6%	10%	0.4	--	--	--	--
			UGCCC	0%	0%	0.8	--	--	--	--
			UGCCG	14%	25%	0.3	6.8	61	0.0599	0.00169
			UGCCU	0%	0%	0.5	--	--	--	--
	L-Ala-pAGCGA	--	--	--	--	0.9	--	--	--	--

Table S3. Yield data of L/D-amino acid-transfer with varied overhangs using phosphoramidate-ester chemistry. Esterification reactions were conducted at room temperature for 18 hours in 50 mM HEPES, 200 mM NaCl, 50 mM $MgCl_2$, 10 mM imidazole, 50 mM EDC hydrochloride pH = 7.0 buffer with 100 μM equal equivalent of donor strand and acceptor strand and 120 μM cytidine as an internal standard. All results from single experiments with exception of those performed with UUCCA overhang which were performed in triplicate and previously reported.

	Donor	Acceptor		Corrected yield
		Stem	Overhang	
**SM-1**	L-Leu-pAGCGA	UCGCU	UGCCA	4.0%
			UACCA	0.7%
			UCCCA	0.9%
			UUCCA	1.9%
			UGACA	6.1%
			UGGCA	8.1%
			UGUCA	6.8%
			UGCAA	0.6%
			UGCUA	1.4%
	D-Leu-pAGCGA	UCGCU	UUCCA	0.2%
	L-Ala-pAGCGA	UCGCU	UUCCA	1.6%
	D-Ala-pAGCGA	UCGCU	UUCCA	0.2%
	L-Val-pAGCGA	UCGCU	UUCCA	1.0%
	D-Val-pAGCGA	UCGCU	UUCCA	0.2%
	L-Ser-pAGCGA	UCGCU	UUCCA	7.2%
	D-Ser-pAGCGA	UCGCU	UUCCA	1.6%
	Gly-pAGCGA	UCGCU	UUCCA	13%
	L-Pro-pAGCGA	UCGCU	UUCCA	0.4%
	L-Arg-pAGCGA	UCGCU	UUCCA	0.8%
	pAGCGA	UCGCU	UUCCA	0%

Table S4. Synthesized oligo(deoxy)ribonucleotides for Next Generation Sequencing experiments. Set a is used for mixed anhydride chemistry; set b is used for phosphoramidate-ester chemistry.

	Sequence (5'-3')	Length
Lib-L5 a	GAAGACGGCAUACGAGAU UCGCU NNNNN	28
Lib-L5 b	[Cy5]GAUAAUACGACUCACAGCAGCGUCCAUCCACA UCGCU NNNNN	42
Lib-S3 a	CAGAAGACGGCAUACGA GAUUCNNN UUCCA	30
Lib-S3 b	[Cy5]GAUAAUACGACUCACAGCAGCGUCCAUCC GAUUCNNN UUCCA	42
Lib-S3-D	pNNNGAAUC	8
Blocker a	ATCTCGTATGCCGTCTT[SpcC3]	17
Ligator a*	App[N]$_{8-12}$AGATCGGAAGAGCGTCGTGTAGGGAAAGAGTGT[SpcC3]	41 – 45
RT primer a<br>Fwd primer a	AATGATACGGCGACCACCGAGATCTACACXXXXXXACACTCTTTCCCTACACGACG	56
Rev primer a	CAAGCAGAAGACGGCATACGAGAT	24
Blocker b	GGATGGACGCTGCTGTGAGTCGTATTATC[SpcC3]	29
Ligator b	pUCUACCUCAUUCUUCACUGGAGACUUGACGAAGCUG[Cy3]	36
RT primer b	TACGCCATTCGAGATCCTCATGCAGCTTCGTCAAGTCTCC	40
Fwd primer b	CAAGCAGAAGACGGCATACGAGATXXXXXXXXGTCTCGTGGGCTCGGAGATGTGTATAAGAGACAGTACGCCATTCG<br>AGATCCTCATGCA	90
Rev primer b	AATGATACGGCGACCACCGAGATCTACACXXXXXXXXTCGTCGGCAGCGTCAGATGTGTATAAGAGACAGGATAATA<br>CGACTCACAGCAGCGTC	94

N, randomized nucleotides; RT, reverse transcription; X, index sequence; *, this DNA oligo is adenylated at its 5'-end through the technique which is being described above using ImpA. Six different oligos with variably long stretches of randomized nucleotides on their 5'-end were used. By this, frameshift between the samples can be achieved, increasing complexity during sequencing which ultimately increases the quality of the generated data. The randomized nucleotides also allow for the identification of PCR duplicates.

Table S5. Rank of quality-filtered read numbers of CCA-ending overhangs from the **Lib-L5** sequencing results (mixed anhydride chemistry). All 4^5 = 1024 overhangs were observed.

	Blank	L-Ala	Gly	Pro	Leu	Val
**UUCCA**	**709**	**98**	**29**	**27**	**63**	**18**
UA**CCA**	761	840	848	815	887	902
AU**CCA**	794	894	927	898	921	1008
AG**CCA**	864	853	1007	963	977	953
AA**CCA**	891	994	985	989	933	983
GG**CCA**	894	822	953	939	952	969
GU**CCA**	895	1005	967	1015	891	1019
UG**CCA**	897	816	1021	968	941	985
CA**CCA**	936	1023	915	873	974	992
AC**CCA**	965	737	863	825	928	910
GA**CCA**	976	922	911	895	962	888
CU**CCA**	985	755	583	741	741	810
UC**CCA**	993	667	482	653	837	967
CG**CCA**	998	1018	1020	1018	1018	1000
CC**CCA**	1022	992	806	897	865	931
GC**CCA**	1024	1017	1003	1002	958	1013

Table S6. Rank of quality-filtered read numbers of CCA-ending overhangs from the **Lib-L5** sequencing results (phosphoramidate-ester chemistry). All 4^5 = 1024 overhangs were observed.

	Blank	L-Ala	Gly	Pro	Leu	Val
**UUCCA**	**707**	**374**	**452**	**461**	**466**	**688**
UA**CCA**	761	324	627	383	188	830
AU**CCA**	800	830	756	665	585	908
AG**CCA**	868	575	682	345	544	676
UG**CCA**	891	269	346	321	139	627
GG**CCA**	895	354	606	182	392	857
GU**CCA**	896	693	887	526	812	913
AA**CCA**	897	951	990	945	765	970
CA**CCA**	941	768	854	405	403	995
AC**CCA**	970	960	992	710	836	1015
GA**CCA**	978	827	949	620	673	952
CU**CCA**	985	250	214	59	229	635
UC**CCA**	989	383	350	206	439	869
CG**CCA**	998	321	240	116	266	832
CC**CCA**	1022	948	885	667	818	1020
GC**CCA**	1024	1009	1023	878	1010	1023

Table S7. Kinetic data of mixed anhydride aminoacyl-transfer with **SM-1**, relating to **Fig. 2A**.

	Donor	Acceptor		Observed yield	Corrected yield	Mixed anhyd. half-life /h	Diol ester half-life /h	Peak time /min	k transfer /min^{-1}	k hydrolysis /min^{-1}
		Stem	Overhang							
**SM-1**	L-Ala-pAGCGA	UCGCU	UUCCA	34%	57%	0.47	10	70	0.0569	0.00116
	L-Ala-pAGCGA	--	--	--	--	0.90	--	--	--	--
	Gly-pAGCGA	UCGCU	UUCCA	2%	3%	0.72	--	--	--	--
	Gly-pAGCGA	--	--	--	--	0.93	--	--	--	--
	L-Val-pAGCGA	UCGCU	UUCCA	4%	10%	0.77	>24	>200	0.0310	<0.00050
	L-Val-pAGCGA	--	--	--	--	0.95	--	--	--	--
	L-Leu-pAGCGA	UCGCU	UUCCA	22%	35%	0.72	16	157	0.0226	0.00073
	L-Leu-pAGCGA	--	--	--	--	0.70	--	--	--	--
	L-Pro-pAGCGA	UCGCU	UUCCA	14%	27%	0.44	2.8	55	0.0492	0.00416
	L-Pro-pAGCGA	--	--	--	--	0.96	--	--	--	--

Table S8. Kinetic data of mixed anhydride aminoacyl-transfer with **SM-2**, relating to **Fig. 2B**.

	Donor	Acceptor		Observed yield	Corrected yield	Mixed anhyd. half-life /h	Diol ester half-life /h	Peak time /min	k transfer /min^{-1}	k hydrolysis /min^{-1}
		Stem	Overhang							
**SM-2**	L-Ala-pAGCAA	UUGCU	UUCCA	15%	54%	0.59	11	65	0.0630	0.00109
	L-Ala-pAGCAA	--	--	--	--	1.27	--	--	--	--
	Gly-pAGCAA	UUGCU	UUCCA	0%	0%	0.73	--	--	--	--
	Gly-pAGCAA	--	--	--	--	1.12	--	--	--	--
	L-Val-pAGCAA	UUGCU	UUCCA	3%	11%	0.98	> 24	> 200	0.0137	< 0.00050
	L-Val-pAGCAA	--	--	--	--	1.65	--	--	--	--
	L-Leu-pAGCAA	UUGCU	UUCCA	21%	36%	0.54	12	136	0.0251	0.00095
	L-Leu-pAGCAA	--	--	--	--	0.76	--	--	--	--
	L-Pro-pAGCAA	UUGCU	UUCCA	21%	29%	0.30	3.9	78	0.0343	0.00296
	L-Pro-pAGCAA	--	--	--	--	0.96	--	--	--	--

Table S9. Kinetic data of mixed anhydride aminoacyl-transfer with **SM-3**, relating to **Fig. 2C**.

	Donor	Acceptor		Observed yield	Corrected yield	Mixed anhyd. half-life /h	Diol ester half-life /h	Peak time /min	k transfer /min^{-1}	k hydrolysis /min^{-1}
		Stem	Overhang							
**SM-3**	L-Ala-pAGUGA	UCACU	UUCCA	17%	30%	0.49	4.2	117	0.0195	0.00273
	L-Ala-pAGUGA	--	--	--	--	1.20	--	--	--	--
	Gly-pAGUGA	UCACU	UUCCA	7%	10%	1.22	> 24	> 200	0.0236	< 0.00050
	Gly-pAGUGA	--	--	--	--	1.63	--	--	--	--
	L-Val-pAGUGA	UCACU	UUCCA	7%	21%	0.59	> 24	> 200	0.0219	< 0.00050
	L-Val-pAGUGA	--	--	--	--	1.02	--	--	--	--
	L-Leu-pAGUGA	UCACU	UUCCA	20%	40%	0.37	11	110	0.0320	0.00105
	L-Leu-pAGUGA	--	--	--	--	0.96	--	--	--	--
	L-Pro-pAGUGA	UCACU	UUCCA	21%	26%	0.45	3.3	69	0.0382	0.00348
	L-Pro-pAGUGA	--	--	--	--	1.01	--	--	--	--

Table S10. Kinetic data of mixed anhydride aminoacyl-transfer with **SM-4**, relating to **Fig. 2D**.

	Donor	Acceptor		Observed yield	Corrected yield	Mixed anhyd. half-life /h	Diol ester half-life /h	Peak time /min	k transfer /min^{-1}	k hydrolysis /min^{-1}
		Stem	Overhang							
**SM-4**	L-Ala-pAGGCA	UGCCU	UUCCA	18%	30%	0.41	6.1	68	0.0501	0.00189
	L-Ala-pAGGCA	--	--	--	--	0.67	--	--	--	--
	Gly-pAGGCA	UGCCU	UUCCA	0%	0%	0.88	--	--	--	--
	Gly-pAGGCA	--	--	--	--	1.61	--	--	--	--
	L-Val-pAGGCA	UGCCU	UUCCA	3%	10%	0.94	> 24	> 200	0.0121	< 0.00050
	L-Val-pAGGCA	--	--	--	--	1.06	--	--	--	--
	L-Leu-pAGGCA	UGCCU	UUCCA	21%	48%	0.37	10	97	0.0369	0.00116
	L-Leu-pAGGCA	--	--	--	--	0.79	--	--	--	--
	L-Pro-pAGGCA	UGCCU	UUCCA	21%	48%	0.35	3.1	44	0.0714	0.00376
	L-Pro-pAGGCA	--	--	--	--	1.17	--	--	--	--

Table S11. Kinetic data of mixed anhydride aminoacyl-transfer with **SM-5**, relating to **Fig. 2E**.

	Donor	Acceptor		Observed yield	Corrected yield	Mixed anhyd. half-life /h	Diol ester half-life /h	Peak time /min	k transfer /min^{-1}	k hydrolysis /min^{-1}
		Stem	Overhang							
**SM-5**	L-Ala-pAACGA	UCGUU	UUCCA	15%	36%	0.54	6.4	79	0.0411	0.00182
	L-Ala-pAACGA	--	--	--	--	0.81	--	--	--	--
	Gly-pAACGA	UCGUU	UUCCA	0%	0%	0.48	--	--	--	--
	Gly-pAACGA	--	--	--	--	0.58	--	--	--	--
	L-Val-pAACGA	UCGUU	UUCCA	5%	16%	0.49	> 24	> 200	0.0199	< 0.00050
	L-Val-pAACGA	--	--	--	--	0.83	--	--	--	--
	L-Leu-pAACGA	UCGUU	UUCCA	15%	33%	0.43	8.0	120	0.0252	0.00145
	L-Leu-pAACGA	--	--	--	--	0.63	--	--	--	--
	L-Pro-pAACGA	UCGUU	UUCCA	20%	31%	0.45	3.5	71	0.0370	0.00334
	L-Pro-pAACGA	--	--	--	--	0.95	--	--	--	--

Table S12. Kinetic data of mixed anhydride aminoacyl-transfer with **SM-6**, relating to **Fig. 2F**.

	Donor	Acceptor		Observed yield	Corrected yield	Mixed anhyd. half-life /h	Diol ester half-life /h	Peak time /min	k transfer /min^{-1}	k hydrolysis /min^{-1}
		Stem	Overhang							
**SM-6**	L-Ala-pCGCAA	UUGCG	UUCCA	34%	52%	1.16	13	156	0.0212	0.00089
	L-Ala-pCGCAA	--	--	--	--	1.06	--	--	--	--
	Gly-pCGCAA	UUGCG	UUCCA	0%	0%	0.71	--	--	--	--
	Gly-pCGCAA	--	--	--	--	1.37	--	--	--	--
	L-Val-pCGCAA	UUGCG	UUCCA	8%	29%	0.53	> 24	> 200	0.0159	< 0.00050
	L-Val-pCGCAA	--	--	--	--	1.18	--	--	--	--
	L-Leu-pCGCAA	UUGCG	UUCCA	43%	60%	0.57	10	128	0.0257	0.00111
	L-Leu-pCGCAA	--	--	--	--	0.94	--	--	--	--
	L-Pro-pCGCAA	UUGCG	UUCCA	23%	36%	0.67	2.2	48	0.0526	0.00531
	L-Pro-pCGCAA	--	--	--	--	1.47	--	--	--	--

Table S13. Quality-filtered read numbers from the sequencing results of **Lib-S3** with the mixed anhydride chemistry.

	Blank	L-Ala	D-Ala	Gly	Pro	Leu	Val
AAA	52	115	71	17	75	28	39
AAC	30	71	76	13	59	23	43
AAG	55	48	122	17	107	58	77
AAU	70	104	182	23	64	18	44
ACA	42	79	76	21	92	40	34
ACC	23	14	33	8	38	8	18
ACG	35	82	79	25	154	48	58
ACU	88	43	142	12	80	18	39
AGA	72	98	64	11	69	28	28
AGC	19	28	76	9	76	37	43
AGG	87	97	148	15	139	86	70
AGU	91	109	213	11	78	52	55
AUC	196	85	39	121	63	16	26
AUG	58	188	145	16	119	67	78
AUU	77	80	130	13	65	28	34
CAA	34	124	67	22	100	35	50
CAC	12	10	44	17	51	17	24
CAG	36	178	83	13	91	40	37
CAU	57	54	154	11	71	33	44
CCA	28	17	50	5	81	19	36
CCC	10	3	26	13	24	5	18
CCG	12	12	49	17	91	26	46
CCU	73	19	65	9	51	18	39
CGA	36	18	31	3	41	30	18
CGC	25	6	33	4	207	22	41
CGG	18	43	42	12	94	43	50
CGU	16	9	38	7	77	40	36
CUA	40	58	64	28	151	65	58
CUC	234	77	30	1349	161	16	26
CUG	54	70	127	108	163	44	65
CUU	65	42	128	15	143	31	52
GAA	69	77	58	43	60	40	57
GAC	28	18	41	21	102	33	43
GAG	121	68	133	99	177	116	108
GAU	110	84	64	17	51	19	35
GCA	62	14	85	29	163	64	67
GCC	28	4	34	18	70	26	49
GCG	81	30	134	137	273	95	111
GCU	96	29	139	34	298	28	132
GGA	80	26	46	32	82	37	42
GGC	30	9	61	9	140	60	52
GGG	133	69	134	35	262	95	109
GGU	90	83	91	8	72	43	52
GUA	116	6	30	9	178	78	45
GUC	23	1	12	17	85	28	43
GUG	176	40	70	29	221	93	79
GUU	40	23	36	10	87	41	61
UAA	69	141	75	15	111	37	64
UAA	83	170	89	33	137	45	93
UAC	48	41	107	19	98	28	53
UAG	64	234	188	30	180	68	114
UAU	79	98	113	24	97	37	56
UCA	52	40	110	22	147	48	69
UCC	56	7	55	4	50	10	23
UCG	86	10	57	11	133	37	67
UCU	179	48	175	95	375	32	66
UGA	93	30	117	55	58	56	67
UGC	150	14	212	49	153	45	78
UGG	59	98	204	62	238	74	96
UGU	149	69	129	29	178	69	61
UUA	75	137	96	22	138	51	78
UUC	47	11	39	16	89	23	45
UUG	82	215	126	15	243	71	107
UUU	111	98	107	29	145	26	59

Table S14. Rankings of quality-filtered read numbers for each triplet and amino acid using **Lib-S3** with the mixed anhydride chemistry.

	L-Ala	Gly	Pro	Val	Leu
AAA	9	35	51	48	47
AAC	25	43	57	44	52
AAG	32	33	29	13	15
AAU	11	23	53	40	58
ACA	22	28	37	55	30
ACC	50	58	63	62	63
ACG	19	21	16	26	20
ACU	35	48	46	49	57
AGA	13	49	49	57	46
AGC	43	56	42	42	35
AGG	15	40	25	14	5
AGU	10	50	43	29	17
AUC	18	3	52	59	61
AUG	3	38	27	11	11
AUU	21	44	54	56	45
CAA	8	24	30	34	36
CAC	55	36	61	60	59
CAG	4	46	38	51	29
CAU	31	51	50	41	38
CCA	49	61	45	52	55
CCC	63	45	64	64	64
CCG	53	34	33	37	50
CCU	46	54	60	50	56
CGA	48	64	62	63	41
CGC	60	63	8	47	53
CGG	34	47	40	35	25
CGU	57	60	44	53	28
CUA	30	20	19	25	12
CUC	24	1	14	58	60
CUG	26	4	15	20	23
CUU	36	39	21	33	40
GAA	23	10	55	27	27
GAC	47	27	32	43	37
GAG	29	5	9	5	1
GAU	17	31	59	54	54
GCA	51	19	13	17	13
GCC	62	30	48	36	49
GCG	40	2	4	3	3
GCU	42	12	2	1	44
GGA	44	14	41	46	33
GGC	58	57	24	32	14
GGG	27	11	3	4	2
GGU	20	59	47	31	24
GUA	61	55	11	38	6
GUC	64	32	39	45	43
GUG	39	16	7	9	4
GUU	45	53	34	23	26
UAA	6	41	28	21	34
UAA	5	13	23	8	22
UAC	37	29	31	30	42
UAG	1	15	12	2	10
UAU	14	22	36	28	32
UCA	38	25	18	15	19
UCC	59	62	58	61	62
UCG	56	52	26	18	31
UCU	33	6	1	19	39
UGA	41	8	56	16	16
UGC	52	9	17	12	21
UGG	12	7	5	7	7
UGU	28	17	10	22	9
UUA	7	26	22	10	18
UUC	54	37	35	39	51
UUG	2	42	6	6	8
UUU	16	18	20	24	48

Table S15. Selectivity scores (see Sequencing and data processing) for each amino acid transferred with **Lib-S3** in the mixed anhydride chemistry. Top 3 of each amino acid are highlighted in red. Trinucleotides selected for synthesis and kinetic verification are highlighted in yellow. A low score indicates high selectivity.

	L-Ala	Gly	Pro	Leu	L&V
AAA	0.050	0.226	0.367	0.329	1.000
AAC	0.128	0.242	0.348	0.308	0.768
AAG	0.356	0.371	0.312	0.140	0.298
AAU	0.063	0.142	0.402	0.457	1.126
ACA	0.147	0.194	0.274	0.211	0.977
ACC	0.203	0.244	0.270	0.270	0.731
ACG	0.229	0.259	0.186	0.244	0.821
ACU	0.175	0.257	0.243	0.320	0.822
AGA	0.065	0.297	0.297	0.274	0.928
AGC	0.246	0.346	0.239	0.191	0.546
AGG	0.179	0.678	0.338	0.053	0.238
AGU	0.072	0.505	0.406	0.129	0.447
AUC	0.103	0.016	0.369	0.462	1.644
AUG	0.034	0.731	0.429	0.139	0.324
AUU	0.106	0.250	0.325	0.257	0.849
CAA	0.065	0.222	0.294	0.375	1.129
CAC	0.255	0.153	0.290	0.278	0.783
CAG	0.024	0.377	0.292	0.209	0.909
CAU	0.172	0.319	0.311	0.220	0.598
CCA	0.230	0.303	0.207	0.266	0.690
CCC	0.266	0.176	0.271	0.271	0.744
CCG	0.344	0.197	0.190	0.318	0.725
CCU	0.209	0.255	0.291	0.267	0.663
CGA	0.209	0.299	0.287	0.173	0.598
CGC	0.351	0.375	0.036	0.298	0.763
CGG	0.231	0.351	0.284	0.160	0.496
CGU	0.308	0.330	0.222	0.131	0.503
CUA	0.395	0.233	0.218	0.128	0.536
CUC	0.180	0.006	0.098	0.619	3.026
CUG	0.419	0.048	0.205	0.354	0.956
CUU	0.271	0.300	0.142	0.310	0.760
GAA	0.193	0.076	0.632	0.235	0.614
GAC	0.338	0.170	0.208	0.248	0.755
GAG	1.450	0.114	0.225	0.021	0.140
GAU	0.086	0.168	0.378	0.335	1.009
GCA	0.823	0.202	0.130	0.130	0.361
GCC	0.380	0.154	0.271	0.278	0.607
GCG	3.333	0.040	0.083	0.061	0.130
GCU	0.712	0.135	0.020	0.772	0.804
GGA	0.328	0.085	0.299	0.228	0.798
GGC	0.457	0.445	0.149	0.082	0.331
GGG	1.350	0.306	0.068	0.044	0.146
GGU	0.124	0.484	0.351	0.153	0.437
GUA	0.555	0.474	0.069	0.036	0.346
GUC	0.403	0.168	0.212	0.239	0.652
GUG	1.083	0.271	0.103	0.056	0.210
GUU	0.331	0.414	0.231	0.168	0.371
UAA	0.048	0.461	0.275	0.354	0.733
UAA	0.076	0.224	0.479	0.449	0.732
UAC	0.280	0.207	0.225	0.331	0.742
UAG	0.026	0.600	0.429	0.333	0.429
UAU	0.119	0.200	0.375	0.320	0.833
UCA	0.494	0.278	0.186	0.198	0.420
UCC	0.243	0.258	0.238	0.258	0.687
UCG	0.441	0.397	0.166	0.204	0.366
UCU	0.508	0.065	0.010	0.661	1.450
UGA	0.427	0.062	0.691	0.132	0.305
UGC	0.881	0.088	0.181	0.233	0.423
UGG	0.462	0.226	0.152	0.226	0.583
UGU	0.483	0.246	0.132	0.117	0.564
UUA	0.092	0.456	0.361	0.277	0.509
UUC	0.333	0.207	0.193	0.309	0.714
UUG	0.032	1.909	0.103	0.143	0.280
UUU	0.145	0.167	0.189	0.615	1.333

Table S16. Quality-filtered read numbers from the sequencing results of phosphoramidate-ester **Lib-S3**.

	Blank	L-Ala	D-Ala	Gly	Pro	Leu	Val
AAA	4725	1735	1962	828	1862	2797	1623
AAC	4634	2274	2805	1078	2535	3661	2007
AAG	5876	4772	5930	2188	5251	7443	4093
AAU	7594	2124	2314	1060	2383	3128	1780
ACA	4234	1582	1703	764	1714	2387	1333
ACC	2328	1133	1508	609	1355	2086	1002
ACG	2748	1842	2282	947	2105	3129	1532
ACU	4821	1816	2270	939	2201	3085	1573
AGA	3174	2233	2316	1019	2355	3789	2082
AGC	7811	5595	6974	2820	6190	9212	4879
AGG	3674	3276	3712	1537	3465	5842	2905
AGU	9447	6560	7770	3198	6817	11166	5661
AUC	4921	1660	1862	707	1775	2601	1398
AUG	4279	1909	2366	815	2167	3128	1567
AUU	5943	3174	3800	1527	3435	5026	2661
CAA	6831	2026	2358	956	2247	3309	1786
CAC	4357	1521	1612	759	1629	2289	1234
CAG	3303	1705	1964	845	1961	2628	1341
CAU	3614	4388	5487	1981	4926	7473	3850
CCA	5689	1812	2148	927	2053	2833	1513
CCC	2108	1173	1294	616	1322	1817	985
CCG	1190	1257	1238	716	1426	1978	1041
CCU	1584	1289	1493	670	1489	2005	1012
CGA	4425	1385	1589	688	1690	2383	1203
CGC	1882	1305	1496	669	1483	2002	1134
CGG	2907	2440	3072	1224	2790	4361	2064
CGU	1807	1255	1296	642	1349	2151	1050
CUA	5078	2209	2720	1079	2446	4020	1918
CUC	3927	1467	1637	730	1635	2491	1261
CUG	2700	1220	1527	606	1392	2308	1039
CUU	3682	2107	2892	1157	2423	3996	1772
GAA	5048	1868	2389	1086	2205	3471	1654
GAC	6257	2712	3128	1230	3032	4510	2307
GAG	4513	5801	5363	2637	6103	9850	4809
GAU	5864	3997	4314	1783	4201	6412	3423
GCA	9055	1935	2361	964	2167	3336	1777
GCC	5908	2210	2729	1079	2508	3897	1772
GCG	2252	2618	3133	1316	3149	4634	2269
GCU	3188	2663	3231	1251	3374	4805	2442
GGA	3162	727	562	361	722	1101	587
GGC	4917	4200	4633	2023	4615	7453	3658
GGG	7810	2267	1828	1011	2297	3444	1783
GGU	4774	3432	3478	1713	3872	6071	3152
GUA	11704	4067	4845	1868	4245	6537	3484
GUC	14869	3159	4268	1520	3584	5484	2580
GUG	7962	5452	6904	2606	6030	9609	4785
GUU	13992	4719	5912	2373	5259	8240	4328
UAA	21102	1884	2157	915	2077	2893	1555
UAA	5740	1782	2311	945	2058	3073	1474
UAC	5108	3647	4606	1778	3953	6151	3171
UAG	4647	2449	2798	1124	2662	3792	2063
UAU	12449	1694	1924	814	1805	2614	1409
UCA	4817	1384	1785	732	1723	2474	1141
UCC	2692	1952	2544	1060	2233	3525	1649
UCG	3215	1871	2224	963	2054	3084	1604
UCU	9417	2056	2270	996	2119	3368	1813
UGA	5909	745	788	363	835	1375	639
UGC	6663	3608	4268	1745	4009	6605	3230
UGG	6216	3824	4302	1763	3889	6764	3338
UGU	22037	1993	2125	953	2076	2991	1663
UUA	5964	2130	2563	1076	2416	3713	1880
UUC	7701	3494	4310	1739	3599	5717	2837
UUG	6467	2562	2870	1271	2645	4257	2171
UUU	10500	2712	3128	1230	3032	4510	2307

Table S17. Rankings of quality-filtered read numbers for each triplet and amino acid in phosphoramidate-ester formation with **Lib-S3**.

	L-Ala	D-Ala	Gly	Pro	Leu	Val
AAA	10	47	47	47	48	47
AAC	45	26	26	30	26	31
AAG	31	5	4	6	6	8
AAU	24	32	37	32	31	39
ACA	6	51	52	50	52	53
ACC	15	62	57	61	60	58
ACG	61	43	39	41	41	38
ACU	14	44	40	43	37	41
AGA	3	28	36	34	32	29
AGC	12	3	2	2	2	4
AGG	26	17	18	17	18	16
AGU	16	1	1	1	1	1
AUC	23	50	49	55	50	50
AUG	11	39	33	48	38	40
AUU	36	18	17	18	19	19
CAA	28	35	35	39	34	37
CAC	20	52	54	51	55	56
CAG	4	48	46	46	47	48
CAU	39	7	6	9	7	6
CCA	35	45	44	44	46	46
CCC	34	61	61	60	62	62
CCG	7	58	62	54	58	61
CCU	60	57	59	57	56	59
CGA	17	54	55	56	53	54
CGC	18	56	58	58	57	60
CGG	21	25	23	24	23	23
CGU	62	59	60	59	61	57
CUA	49	30	29	28	28	25
CUC	32	53	53	53	54	51
CUG	8	60	56	62	59	55
CUU	47	33	24	25	29	26
GAA	38	42	32	27	36	33
GAC	43	9	16	7	11	9
GAG	57	20	22	23	22	22
GAU	1	2	7	3	3	2
GCA	2	11	11	11	10	13
GCC	5	38	34	37	39	36
GCG	51	29	28	29	27	27
GCU	56	22	21	20	21	21
GGA	59	21	20	22	20	20
GGC	37	64	64	64	64	64
GGG	53	8	9	8	8	7
GGU	64	27	50	35	33	34
GUA	58	16	19	16	15	15
GUC	42	10	8	10	9	12
GUG	54	19	14	19	17	18
GUU	63	4	3	4	4	3
UAA	52	6	5	5	5	5
UAA	25	40	43	45	42	45
UAC	9	46	38	42	44	43
UAG	46	13	10	12	13	14
UAU	29	24	27	26	24	28
UCA	13	49	48	49	49	49
UCC	22	55	51	52	51	52
UCG	48	37	31	33	35	32
UCU	27	41	42	38	45	42
UGA	40	34	41	36	40	35
UGC	33	63	63	63	63	63
UGG	55	14	15	14	12	11
UGU	44	12	13	13	14	10
UUA	19	36	45	40	43	44
UUC	30	31	30	31	30	30
UUG	50	15	12	15	16	17
UUU	41	23	25	21	25	24

Table S18. Kinetic data of mixed anhydride aminoacyl-transfer with **SM-11**, relating to **Fig. 3A**. The yield in bold indicates the highest yield among all the amino acid transfers. Shading line indicates a G:U wobble basepair.

	Donor	Acceptor		Observed yield	Corrected yield	Mixed anhyd. half-life /h	Diol ester half-life /h	Peak time /min	k transfer /min^{-1}	k hydrolysis /min^{-1}
		Stem	Overhang							
**SM-11**	**L-Ala-pCUGCG**	CGCAG	UUCCA	**49%**	**61%**	0.71	7.5	63	0.0591	0.00154
	L-Ala-pCUGCG	CGUAG	UUCCA	28%	34%	/	14	135	0.0268	0.00081
	L-Ala-pCUGCG		--	--	--	1.23	--	--	--	--
	D-Ala-pCUGCG	CGCAG	UUCCA	8%	15%	0.46	20	207	0.0170	0.00057
	D-Ala-pCUGCG		--	--	--	1.52	--	--	--	--
	Gly-pCUGCG	CGCAG	UUCCA	0%	0%	/	--	--	--	--
	Gly-pCUGCG		--	--	--	/	--	--	--	--
	L-Val-pCUGCG	CGCAG	UUCCA	14%	24%	1.11	> 24	> 200	0.0170	< 0.00050
	L-Val-pCUGCG		--	--	--	1.48	--	--	--	--
	L-Leu-pCUGCG	CGCAG	UUCCA	28%	45%	0.40	7.7	65	0.0573	0.00151
	L-Leu-pCUGCG		--	--	--	0.90	--	--	--	--
	L-Pro-pCUGCG	CGCAG	UUCCA	29%	36%	0.31	2.2	51	0.0494	0.00523
	L-Pro-pCUGCG		--	--	--	1.50	--	--	--	--

/, not measured.

Table S19. Kinetic data of mixed anhydride aminoacyl-transfer with **SM-13**, relating to **Fig. 3B**.

	Donor	Acceptor		Observed yield	Corrected yield	Mixed anhyd. half-life /h	Diol ester half-life /h	Peak time /min	k transfer /min^{-1}	k hydrolysis /min^{-1}
		Stem	Overhang							
**SM-13**	L-Ala-pUACCG	CGGUA	UUCCA	14%	18%	0.90	4.8	36	0.1084	0.00243
	L-Ala-pUACCG	--		--	--	1.14	--	--	--	--
	D-Ala-pUACCG	CGGUA	UUCCA	15%	17%	0.39	6.4	79	0.0413	0.00179
	D-Ala-pUACCG	--		--	--	0.41	--	--	--	--
	Gly-pUACCG	CGGUA	UUCCA	2%	2%	0.73	--	--	--	--
	Gly-pUACCG	--		--	--	1.27	--	--	--	--
	L-Val-pUACCG	CGGUA	UUCCA	0%	0%	0.70	--	--	--	--
	L-Val-pUACCG	--		--	--	0.72	--	--	--	--
	**L-Leu-pUACCG**	CGGUA	UUCCA	**34%**	**50%**	0.76	18	166	0.0220	0.00063
	L-Leu-pUACCG	--		--	--	0.99	--	--	--	--
	L-Pro-pUACCG	CGGUA	UUCCA	22%	37%	0.52	4.2	74	0.0379	0.00277
	L-Pro-pUACCG	--		--	--	1.30	--	--	--	--

Table S20. Kinetic data of mixed anhydride aminoacyl-transfer with **SM-14**, relating to **Fig. 3C**.

	Donor	Acceptor		Observed yield	Corrected yield	Mixed anhyd. half-life /h	Diol ester half-life /h	Peak time /min	k transfer /min^{-1}	k hydrolysis /min^{-1}
		Stem	Overhang							
**SM-14**	L-Ala-pCUCCG	CGGAG	UUCCA	31%	33%	0.43	7.0	97	0.0326	0.00165
	L-Ala-pCUCCG		--	--	--	1.36	--	--	--	--
	D-Ala-pCUCCG	CGGAG	UUCCA	14%	15%	1.01	7.7	137	0.0207	0.00150
	D-Ala-pCUCCG		--	--	--	1.31	--	--	--	--
	Gly-pCUCCG	CGGAG	UUCCA	7%	8%	/	17	54	0.0912	0.00068
	Gly-pCUCCG		--	--	--	2.06	--	--	--	--
	**L-Val-pCUCCG**	CGGAG	UUCCA	**6%**	**32%**	0.90	> 24	> 200	0.0130	< 0.00050
	L-Val-pCUCCG		--	--	--	1.58	--	--	--	--
	**L-Leu-pCUCCG**	CGGAG	UUCCA	**29%**	**51%**	0.65	16	135	0.0278	0.00072
	L-Leu-pCUCCG		--	--	--	1.44	--	--	--	--
	L-Pro-pCUCCG	CGGAG	UUCCA	21%	30%	0.68	2.6	84	0.0249	0.00445
	L-Pro-pCUCCG		--	--	--	1.89	--	--	--	--

Table S21. Kinetic data of mixed anhydride aminoacyl-transfer with **SM-15**, relating to **Fig. 3D**.

	Donor	Acceptor		Observed yield	Corrected yield	Mixed anhyd. half-life /h	Diol ester half-life /h	Peak time /min	k transfer /min^{-1}	k hydrolysis /min^{-1}
		Stem	Overhang							
**SM-15**	L-Ala-pAGACG	CGUCU	UUCCA	22%	23%	0.47	6.5	123	0.0223	0.00177
	L-Ala-pAGACG		--	--	--	1.43	--	--	--	--
	D-Ala-pAGACG	CGUCU	UUCCA	15%	17%	0.74	3.2	145	0.0119	0.00356
	D-Ala-pAGACG		--	--	--	1.65	--	--	--	--
	Gly-pAGACG	CGUCU	UUCCA	0%	0%	1.05	--	--	--	--
	Gly-pAGACG		--	--	--	1.00	--	--	--	--
	L-Val-pAGACG	CGUCU	UUCCA	4%	7%	0.74	> 24	> 200	0.0174	< 0.00050
	L-Val-pAGACG		--	--	--	1.48	--	--	--	--
	L-Leu-pAGACG	CGUCU	UUCCA	14%	25%	0.44	> 24	> 200	0.0218	< 0.00050
	L-Leu-pAGACG		--	--	--	1.48	--	--	--	--
	**L-Pro-pAGACG**	CGUCU	UUCCA	**24%**	**32%**	0.42	3.1	65	0.0405	0.00375
	L-Pro-pAGACG		--	--	--	0.96	--	--	--	--

Table S22. Kinetic data of mixed anhydride aminoacyl-transfer with **SM-12**, relating to **Fig. 3E**.

	**Donor**	**Acceptor**		**Observed yield**	**Corrected yield**	**Mixed anhyd. half-life** /h	**Diol ester half-life** /h	**Peak time** /min	**k transfer** /min^{-1}	**k hydrolysis** /min^{-1}
		**Stem**	**Overhang**							
**SM-12**	L-Ala-pGAGCG	CGCUC	UUCCA	18%	21%	0.48	27	141	0.0308	0.00043
	L-Ala-pGAGCG	--		--	--	1.52	--	--	--	--
	D-Ala-pGAGCG	CGCUC	UUCCA	11%	13%	0.93	24	150	0.0273	0.00048
	D-Ala-pGAGCG	--		--	--	1.86	--	--	--	--
	**Gly-pGAGCG**	CGCUC	UUCCA	**9%**	**12%**	0.32	> 24	74	0.0732	< 0.00050
	Gly-pGAGCG	--		--	--	1.67	--	--	--	--
	L-Val-pGAGCG	CGCUC	UUCCA	1%	7%	0.61	> 24	> 200	0.0394	< 0.00050
	L-Val-pGAGCG	--		--	--	0.65	--	--	--	--
	L-Leu-pGAGCG	CGCUC	UUCCA	10%	23%	0.58	> 24	190	0.0228	< 0.00050
	L-Leu-pGAGCG	--		--	--	1.67	--	--	--	--
	L-Pro-pGAGCG	CGCUC	UUCCA	12%	16%	0.63	6.1	98	0.0302	0.00189
	L-Pro-pGAGCG	--		--	--	2.06	--	--	--	--

Table S23. Kinetic data of mixed anhydride aminoacyl-transfer with **SM-16** and **SM-17**, relating to **Fig. 3F** and **3G**.

	Donor	Acceptor		Observed yield	Corrected yield	Mixed anhyd. half-life /h	Diol ester half-life /h	Peak time /min	k transfer /min^{-1}	k hydrolysis /min^{-1}
		Stem	Overhang							
**SM-16**	**L-Ala-pCUGC**	CGCAG	UUCCA	**48%**	**62%**	/	6.6	63	0.0573	0.00175
	L-Ala-pCUGC		--	--	--	0.43	--	--	--	--
	L-Pro-pCUGC	CGCAG	UUCCA	26%	35%	/	1.8	50	0.0456	0.00631
	L-Pro-pCUGC		--	--	--	1.12	--	--	--	--
	L-Leu-pCUGC	CGCAG	UUCCA	27%	46%	0.41	14	97	0.0408	0.00083
	L-Leu-pCUGC		--	--	--	0.56	--	--	--	--

M-17 Donor	cceptor		Temp.	bserved yield	orrected yield	Mixed anhyd. half-life /h	Diol ester half-life /h	Peak time /min	k transfer /min^{-1}	k hydrolysis /min^{-1}	Donor eq.
	tem	verhang	C								
L-Ala-pCUG	CGCAG	UUCCA	5	37%	44%	/	13	88	0.0458	0.00089	1 eq.
**L-Ala-pCUG**	CGCAG	UUCCA	**10**	**38%**	**46%**	/	8.3	115	0.0272	0.00140	1 eq.
L-Ala-pCUG	CGCAG	UUCCA	16	23%	27%	/	6.1	71	0.0470	0.00190	1 eq.
L-Ala-pCUG	CGCAG	UUCCA	16	43%	52%	/	4.8	74	0.0404	0.00239	3 eq.
L-Ala-pCUG		--	10	--	--	0.63	--	--	--	--	
L-Pro-pCUG	CGCAG	UUCCA	10	17%	20%	/	3.1	88	0.0255	0.00373	1 eq.
L-Pro-pCUG	CGCAG	UUCCA	16	13%	14%	/	2.7	75	0.0304	0.00423	1 eq.
L-Pro-pCUG		--	10	--	--	1.20	--	--	--	--	
L-Leu-pCUG	CGCAG	UUCCA	10	19%	23%	0.69	26	172	0.0234	0.00045	1 eq.
L-Leu-pCUG	CGCAG	UUCCA	16	11%	12%	0.44	17	103	0.0398	0.00069	1 eq.
L-Leu-pCUG		--	10	--	--	0.66	--	--	--	--	

Table S24. Kinetic data of mixed anhydride aminoacyl-transfer with **SM-18** (triplet sequence for best L-Ala transfer yield), and **SM-19**.

	Donor	Acceptor		Observed yield	Corrected yield	Mixed anhyd. half-life /h	Diol ester half-life /h	Peak time /min	k transfer $/min^{-1}$	k hydrolysis $/min^{-1}$
		Stem	Overhang							
**SM-18**	L-Ala-pCUAAC	GUUAC	UUCCA	40%	51%	0.37	4.0	42	0.0829	0.00288
	L-Ala-pCUAAC	--	--	--	--	0.63	--	--	--	--
**SM-19**	L-Leu-pACUUG	CAAGU	UUCCA	15%	25%	1.05	8.0	> 200	0.0105	0.0014
	L-Leu-pACUUG	--	--	--	--	1.19	--	--	--	--

Table S25. Overview of duplexes for which simulations were conducted in **Fig. 4**, **Fig. S8** and **S9**. Energy landscapes and characteristic structures for these systems are publicly available from the zenodo repository.

	Donor	Acceptor		Corrected	k transfer
		Stem	Overhang	yield	/min^{-1}
**SM-18**	L-Ala-pCUAAC	5′-GUUAG	UUCCA-3′	51%	0.0829
**SM-4**	L-Ala-pAGGCA	5′-UGCCU	UUCCA-3′	30%	0.0501
**SM-6**	L-Leu-pCGCAA	5′-UUGCG	UUCCA-3′	60%	0.0257
**SM-19**	L-Leu-pACUUG	5′-CAAGU	UUCCA-3′	25%	0.0105
**SM-11**	L-Ala-pCUGCG	5′-CGCAG	UUCCA-3′	61%	0.0591
**SM-11**	D-Ala-pCUGCG	5′-CGCAG	UUCCA-3′	15%	0.0170
**SM-11**	L-Leu-pCUGCG	5′-CGCAG	UUCCA-3′	45%	0.0573
**SM-11**	L-Val-PCUGCG	5′-CGCAG	UUCCA-3′	24%	0.0170
**SM-13**	L-Ala-pUACCG	5′-CGGUA	UUCCA-3′	18%	0.1084
**SM-13**	D-Ala-pUACCG	5′-CGGUA	UUCCA-3′	17%	0.0413
**SM-13**	L-Leu-pUACCG	5′-CGGUA	UUCCA-3′	50%	0.0220
**SM-13**	L-Val-pUACCG	5′-CGGUA	UUCCA-3′	0%	--

Reference

1. J. A. Joseph, K. Roder, D. Chakraborty, R. G. Mantell, D. J. Wales, Exploring biomolecular energy landscapes. *Chem Commun (Camb)* **53**, 6974-6988 (2017).
2. K. Röder, J. A. Joseph, B. E. Husic, D. J. Wales, Energy Landscapes for Proteins: From Single Funnels to Multifunctional Systems. *Advanced Theory and Simulations* **2**, (2019).
3. A. Perez *et al.*, Refinement of the AMBER force field for nucleic acids: improving the description of alpha/gamma conformers. *Biophys J* **92**, 3817-3829 (2007).
4. M. Zgarbova *et al.*, Refinement of the Cornell et al. Nucleic Acids Force Field Based on Reference Quantum Chemical Calculations of Glycosidic Torsion Profiles. *J Chem Theory Comput* **7**, 2886-2902 (2011).
5. C. Tian *et al.*, ff19SB: Amino-Acid-Specific Protein Backbone Parameters Trained against Quantum Mechanics Energy Surfaces in Solution. *J Chem Theory Comput* **16**, 528-552 (2020).
6. G. W. T. M. J. Frisch, *et al.*, Gaussian, Inc. (2004).
7. C. I. Bayly, P. Cieplak, W. Cornell, P. A. Kollman, A well-behaved electrostatic potential based method using charge restraints for deriving atomic charges: the RESP model. *The Journal of Physical Chemistry* **97**, 10269-10280 (1993).
8. M. Popenda *et al.*, Automated 3D structure composition for large RNAs. *Nucleic Acids Res* **40**, e112 (2012).
9. D. J. Wales, J. P. K. Doye, Global Optimization by Basin-Hopping and the Lowest Energy Structures of Lennard-Jones Clusters Containing up to 110 Atoms. *The Journal of Physical Chemistry A* **101**, 5111-5116 (1997).
10. O. M. Becker, M. Karplus, The topology of multidimensional potential energy surfaces: Theory and application to peptide structure and kinetics. *The Journal of Chemical Physics* **106**, 1495-1517 (1997).
11. D. J. Wales, M. A. Miller, T. R. Walsh, Archetypal energy landscapes. *Nature* **394**, 758-760 (1998).
12. E. F. Pettersen *et al.*, UCSF Chimera--a visualization system for exploratory research and analysis. *J Comput Chem* **25**, 1605-1612 (2004).
